# Vacancy‐Driven Ni Exsolution in Prussian Blue Analogues Creates Cooperative Defect–Metal Sites for Alkaline Hydrogen Evolution

**DOI:** 10.1002/advs.76307

**Published:** 2026-07-03

**Authors:** Shiqi Wang, Haixian Yan, Hugo L. S. Santos, Md Mofakkharulhashan, Wenyi Huo, Pedro H. C. Camargo

**Affiliations:** ^1^ Department of Chemistry University of Helsinki Helsinki Finland; ^2^ NOMATEN Centre of Excellence National Centre for Nuclear Research Otwock Poland

**Keywords:** electrocatalysis, hydrogen evolution reaction, metal‐exsolution, multimetallic Prussian blue analogues, tuned electronic structure

## Abstract

Alkaline hydrogen evolution reaction (HER) is limited by slow water dissociation and by catalysts that degrade in saline electrolytes. Here we program vacancies to trigger selective Ni exsolution in multimetallic Prussian blue analogues (PBAs), creating cooperative defect–metal interfaces. Low‐temperature annealing of FeMn@CoNi PBAs forms hollow nanocages (PBA‐350) rich in cyanide vacancies and decorated with in situ exsolved Ni nanoparticles. Operando XRD/XAS, operando impedance, and theory reveal a dual‐site mechanism: vacancy‐stabilized Ni lowers the Volmer barrier, adjacent Co facilitates OH* removal, and the vacancy‐modified lattice tunes H* binding toward thermoneutrality. PBA‐350 delivers 28.4 mV at 10 mA cm^−2^ and a 56 mV dec^−1^ Tafel slope in 1.0 m KOH with negligible degradation over 100 h at −50 mA cm^−2^. An anion‐exchange membrane electrolyzer reaches 1.76 V at 1.0 A cm^−2^, and PBA‐350 remains stable in simulated seawater (1.0 m KOH + 0.5 m NaCl) by physically repelling chloride ions via hydration layers, establishing vacancy‐assisted exsolution as a design rule for HER.

## Graphical abstract

Multimetal Prussian blue analogues (PBAs) are converted by low‐temperature Ar annealing into a cavity/CN‐vacancy architecture that triggers in situ Ni exsolution, creating dense Ni‐PBA interfacial motifs. These motifs optimize water dissociation and H* adsorption in alkaline media, delivering markedly accelerated HER kinetics and long‐term stability. When integrated into an anion‐exchange‐membrane (AEM) electrolyzer, the optimized PBA catalysts enable outstanding cell performance.

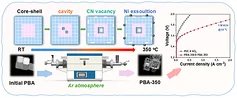

## 1 Introduction

Green hydrogen is central to decarbonizing chemicals, fuels and heavy industry, yet large‐scale deployment is constrained by the efficiency and durability of water electrolysis [1, 2, 3]. At present, low‐emission hydrogen accounts for less than 1% of global hydrogen production, highlighting the persistent gap between climate targets and practical hydrogen deployment [3]. Alkaline and anion‐exchange membrane water electrolyzers offer lower system cost and broader materials choices than proton‐exchange systems, but alkaline hydrogen evolution is intrinsically sluggish because it requires water dissociation [4, 5, 6, 7, 8]. Since the cost of electrolytic hydrogen is strongly linked to electricity price, electrolyzer efficiency, catalyst loading, and operational lifetime, earth‐abundant catalysts that operate efficiently at ≥0.5–1.0 A cm^−2^ are directly relevant to reducing practical hydrogen‐production costs. Meanwhile, industrial operation requires long‐term stability under concentrated alkaline and impure‐water conditions [2, 3]. As a result, even Pt‐based catalysts lose activity and utilization in alkaline environments, while their cost limits gigawatt‐scale deployment [9]. Therefore, resolving the industrial bottlenecks of low energy efficiency, catalyst cost, and durability under device‐relevant conditions is essential for translating laboratory hydrogen evolution reaction (HER) catalysts into practical electrolyzer systems. These challenges motivate earth‐abundant HER catalysts that combine high intrinsic kinetics with long‐term stability in both alkaline and saline electrolytes under device‐relevant current densities.

Prussian blue analogues (PBAs) provide an attractive platform to engineer such catalysts [10, 11]. Their cyanide‐bridged frameworks host multiple transition metals in ordered coordination environments, enabling systematic control over local geometry, redox chemistry and electronic structure [12]. Accordingly, PBAs and PBA‐derived materials have emerged as versatile platforms for HER electrocatalysis because their tunable metal nodes, open frameworks and conversion chemistry allow the construction of catalytically active metal–ligand and metal–compound interfaces. They can serve directly as multimetallic catalytic scaffolds or be converted into metal phosphides, sulfides, carbides, alloys and heteroatom‐doped carbon composites, which improves conductivity, increases active‐site exposure and tunes the adsorption energetics of hydrogen‐ and water‐related intermediates [11]. Thermal transformation can introduce porosity, vacancies and lattice strain [13, 14], while multimetallic PBAs can undergo selective metal exsolution to generate dispersed metallic nanoparticles coupled to the parent lattice [15]. Despite these advances, pristine PBAs are often limited by poor charge transport and incomplete utilization of framework metal nodes, whereas harsh thermal derivatization can cause framework collapse, metal aggregation and loss of well‐defined coordination motifs [12]. More importantly, how CN‐vacancy formation, framework reconstruction and metal exsolution co‐evolve, and how this coupling controls alkaline HER kinetics and saline‐electrolyte durability, remains poorly understood. Thus, a mild reconstruction route capable of preserving the structural advantages of PBAs while creating conductive, defect‐rich and interface‐abundant active motifs is needed. A predictive strategy to program cooperative defect–metal motifs in PBAs is still lacking.

Here we show that controlled low‐temperature annealing of core–shell FeMn@CoNi PBAs produces hollow nanocages (PBA‐350) enriched with cyanide vacancies and decorated with in situ exsolved Ni nanoparticles, generating a high density of defect–metal interfaces. Operando measurements and theory reveal a dual‐site mechanism in which vacancy‐stabilized Ni accelerates water dissociation, adjacent Co facilitates OH* removal, and vacancy‐tuned metal–ligand covalency brings hydrogen adsorption toward thermoneutrality. The resulting PBA‐350 catalyst delivers an overpotential of 28.4 mV at 10 mA cm^−2^ and a Tafel slope of 56 mV dec^−1^ in 1.0 m KOH, approaching commercial Pt/C under matched alkaline conditions while relying solely on earth‐abundant metals. It sustains −50 mA cm^−2^ for 100 h and maintains high activity in alkaline simulated seawater. Integrated into an anion‐exchange membrane water electrolyzer (AEMWE), PBA‐350 achieves 1.76 V at 1.0 A cm^−2^ and exhibits stable operation under continuous load, directly addressing the key industrial requirements of low noble‐metal dependence, reduced energy loss, and stable operation at practically relevant current density. These results establish vacancy‐assisted metal exsolution in PBAs as a design principle for cooperative electrocatalysts that bridge atomic‐scale interface engineering with device‐level hydrogen production.

## 2 Results and Discussion

To test whether vacancy‐driven Ni exsolution in multimetallic Prussian blue analogues can create cooperative defect–metal sites for alkaline hydrogen evolution, we prepared a series of thermally transformed FeMn@CoNi PBAs. We first map how the core–shell framework evolves under low‐temperature annealing, focusing on the emergence of hollow architectures, cyanide vacancies, and Ni nanoparticles. We then connect these structural changes to element‐specific electronic rearrangements, alkaline HER activity and durability, and finally to AEMWE and alkaline seawater electrolysis performance.

### 2.1 Synthesis and Characterization

FeMn@CoNi PBA nanocubes (denoted PBA‐RT) under Ar (Figure 1a). The FeMn@CoNi precursor, synthesized following our previous work [15, 16], consists of a FeMn‐rich core and a CoNi‐rich shell with well‐defined cubic morphology and narrow size distribution. Thermogravimetric analysis reveals a multistep transformation, with dehydration below 150°C, the onset of framework degradation and cavity formation around 200°C, progressive CN vacancy generation above 200°C, and the emergence of metallic nanoparticles beyond 300°C (Figure S1). To capture these stages, a series of samples annealed at 200°C, 300°C, 350°C, and 450°C were prepared and labeled PBA‐200, PBA‐300, PBA‐350, and PBA‐450, respectively.

**FIGURE 1:**
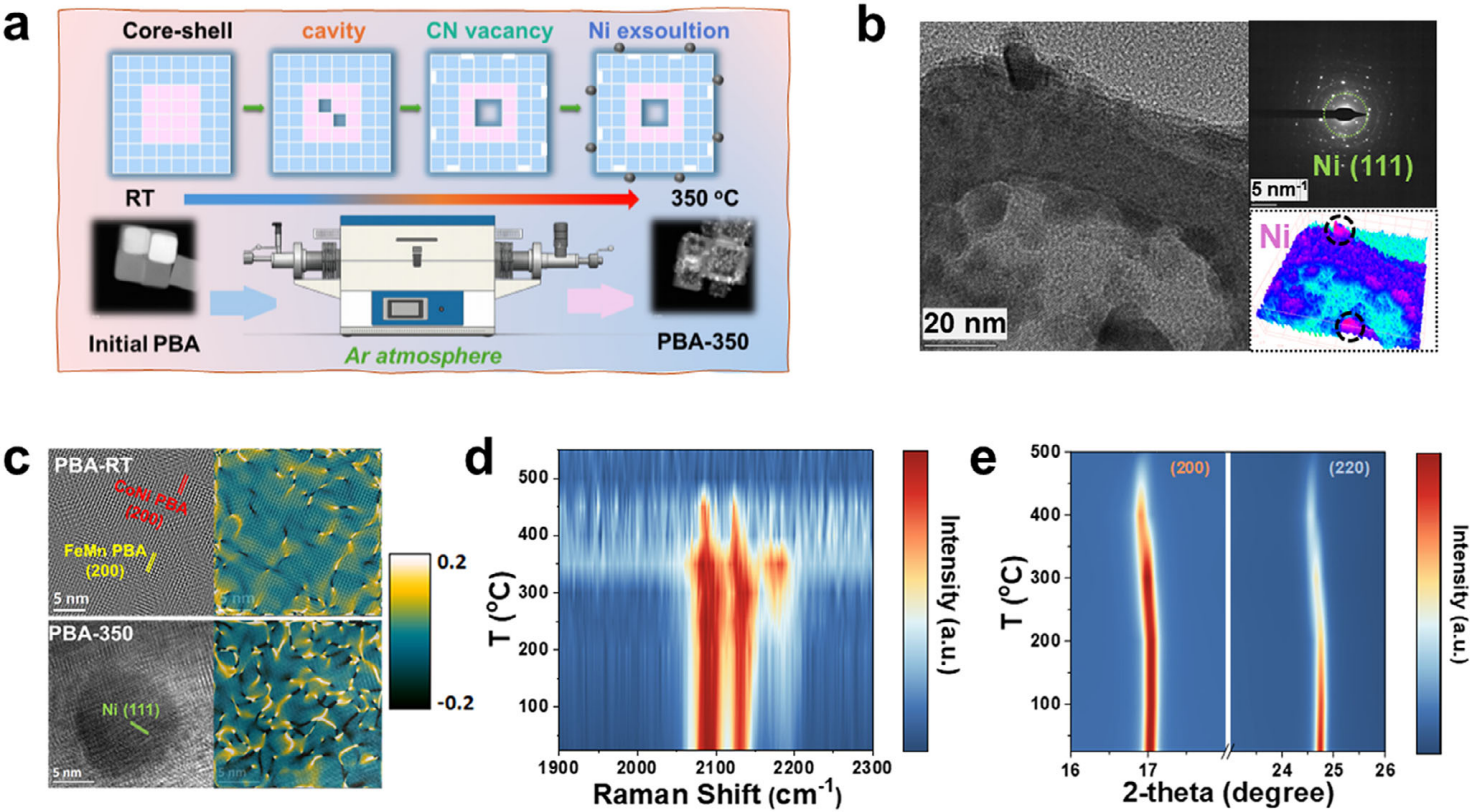
Vacancy‐driven Ni exsolution and hollow nanocage formation in multimetallic PBAs. (a) Schematic illustration of the thermally transformed PBA‐350 nanocage, showing a hollow FeMn@CoNi Prussian blue analogue (PBA) framework decorated with exsolved Ni nanoparticles and cyanide vacancies. (b) High‐resolution TEM image and corresponding 3D surface plot of a PBA‐350 nanocube, with the inset selected‐area electron diffraction (SAED) pattern evidencing a polycrystalline yet ordered framework. (c) Geometric phase analysis of PBA‐RT and PBA‐350 highlighting the build‐up of elastic strain upon Ni exsolution and hollowing. (d) Raman spectra of PBA‐derived samples annealed at different temperatures, revealing the progressive attenuation of Fe–CN–Mn bands and the evolution of Co–CN–Ni signatures during framework softening and vacancy formation. (e) In situ synchrotron XRD patterns of PBA‐RT collected from room temperature to 500°C, showing lattice expansion, peak broadening, and the emergence of Ni (111) reflections.

Scanning electron microscopy shows that PBA‐RT consists of uniform solid cubes with a smooth surface, while the overall cubic framework is preserved up to 350°C (Figures S2 and S3). The morphology transformation could also lead to a difference in physicochemical properties, such as the optical property (Figure S4) [17, 18, 19, 20]. The corresponding STEM–EDS maps confirm the compositional complexity and core–shell architecture of PBA‐RT, with Fe/Mn enriched in the core and Co/Ni in the shell (Figures S5–S7). As the annealing temperature is raised to 200°C and 300°C, internal voids develop within the cubes and gradually expand, whereas the external contours remain largely intact (Figures S8–S11). This behavior is consistent with the lower thermal stability of the FeMn domains, which decompose first and generate internal cavities that later evolve into a hollow framework [21].

At 350°C, the precursor evolves into well‐defined hollow nanocages (PBA‐350) while retaining the macroscopic cubic shape (Figure 1b; Figures S12 and S13). High‐resolution TEM and HAADF‐STEM images reveal thin shells decorated with uniformly dispersed nanoparticle islands of ≈10 nm, which are enriched in Ni as shown by EDS mapping. Selected‐area electron diffraction patterns display weak rings associated with metallic Ni in addition to broadened PBA reflections, indicating partial exsolution of Ni from the CoNi shell. Geometric phase analysis highlights pronounced elastic strain in PBA‐350 compared with PBA‐RT (Figure 1c), arising from lattice mismatch between the exsolved Ni nanoparticles and the surrounding cyanide framework [22, 23]. This strain perturbs the local coordination environment and is expected to modulate the electronic structure at the defect–metal interface [24, 25].

Raman spectroscopy was used to track the evolution of cyanide coordination and vacancy formation during annealing. All samples display four main bands between 2000 and 2250 cm^−^^1^ characteristic of cyanide stretching in PBAs (Figure 1d; Figure S17). The dominant peaks near 2090 and 2130 cm^−^^1^ are assigned to Fe–CN–Mn linkages [26], whereas the higher‐energy bands originate from Co–CN–Ni units. With increasing temperature, Fe–CN–Mn signals progressively weaken and broaden before sharpening again at 350°C, consistent with the decomposition of unstable interior domains to generate cavities followed by partial framework reconstruction. In contrast, Co–CN–Ni bands initially intensify and then diminish sharply above 350°C, disappearing almost completely at 450°C. Similar trends are observed in the low‐frequency region (100–400 cm^−^^1^, Figure S18). These spectral changes indicate that Ni exsolution is intimately coupled to CN bond cleavage and vacancy formation within the CoNi shell.

In situ synchrotron X‐ray diffraction (SXRD) provides direct evidence for the thermally driven transformation of the PBA lattice (Figure 1e; Figure S19). As the temperature increases from room temperature to 500°C, the low‐angle reflections such as (200) and (220) shift gradually to lower angles, signaling lattice expansion and framework softening associated with dehydration [27, 28], defect formation, and strain release. Concurrently, the reflections broaden and lose intensity, reflecting increasing disorder and loss of long‐range crystallinity. The (422) PBA reflection diminishes and disappears above 350°C–400°C, while a new peak corresponding to Ni (111) emerges and grows. Together with ex situ SXRD and elemental analysis (Figures S20 and S21), these results establish a transition from an ordered cyanide‐bridged framework to a defect‐rich hollow architecture decorated with exsolved Ni nanoparticles at 350°C [16], followed by complete framework collapse and extensive metal crystallization at higher temperature (PBA‐450 and above). Importantly, no crystalline oxide phase is detected at 450°C, although PBAs commonly transform into metal oxides when heated in air. This difference is attributed to the Ar atmosphere used here, which suppresses oxidative decomposition of the cyanide framework. Thus, the transformation proceeds through an inert‐atmosphere vacancy–exsolution pathway rather than a PBA‐to‐oxide phase transition. Three independent observations in the present data confirm this assignment. In situ and ex situ SXRD (Figure 1e; Figures S19 and S20) show the disappearance of PBA reflections and the emergence of Ni (111) with no detectable oxide reflections at any temperature. Raman spectroscopy (Figure 1d) shows progressive loss of CN‐stretching modes rather than the appearance of metal‐oxide vibrational bands. TEM, SAED, and HRTEM of PBA‐450 reveal framework collapse and coarsened metallic particles, not oxide nanocrystals.

Electron paramagnetic resonance (EPR) spectroscopy provides direct evidence for vacancy‐associated paramagnetic centers generated during thermal reconstruction. As shown in Figure S22, the signal at g ≈ 2.077 intensifies progressively from PBA‐RT to PBA‐200 and PBA‐350, tracking the gradual accumulation of unpaired electronic states at undercoordinated metal‐cyanide sites. CN removal generates locally undercoordinated metal‐cyanide environments that perturb the framework electronic structure, so the enhanced EPR response provides a direct paramagnetic‐center fingerprint of vacancy formation. This trend is consistent with three independent lines of evidence already established above: the Raman attenuation of CN‐stretching modes (Figure 1d), the lattice expansion and peak broadening from in situ SXRD (Figure 1e), and the element‐specific valence changes and local coordination reconstruction revealed by X‐ray spectroscopy (discussed in the following section). The concurrent emergence of Ni exsolution further indicates that vacancy generation and selective Ni release proceed in a coupled fashion, with the defect‐rich framework stabilizing the exsolved Ni species. Comparative experiments on ternary PBAs annealed at 350°C show that hollow structures with metal exsolution form only when both Ni‐containing and FeMn‐containing domains are present (Figures S23–S25), confirming the cooperative roles of Ni in driving metal release and FeMn in templating cavity formation. The role of vacancies is isolated here through three internally consistent control layers. First, the temperature‐dependent annealing series (PBA‐RT, PBA‐200, PBA‐300, PBA‐350, PBA‐450), derived from the identical FeMn@CoNi precursor, varies only the thermal driving force, allowing the joint evolution of CN‐vacancy density, Ni exsolution, ECSA, and HER activity to be tracked along a single reconstruction coordinate. Second, the compositional control (Figures S23–S25) shows that hollow architectures with metal exsolution form only when both Ni‐containing and FeMn‐containing domains are present. Third, theoretical modeling of pristine PBA, vacancy‐containing PBA‐VCN, and Ni‐decorated Ni/PBA‐VCN surfaces deconvolutes the electronic effect of CN vacancies from that of Ni decoration, as detailed in the mechanistic analysis below. External Ni deposition on unannealed PBAs would require reductive or coordination‐altering conditions that themselves perturb the cyanide framework and introduce additional defects, making a clean Ni‐only control impossible by construction. Taken together, these three control layers jointly identify the vacancy‐rich Ni/PBA interfacial motif, rather than Ni exsolution alone or CN vacancies alone, as the origin of the enhanced alkaline HER activity, and confirm that a concerted reconstruction at 350°C is required to generate the cooperative defect–metal interface responsible for the Pt‐like kinetics of PBA‐350. The increased BET surface area further supports the improved accessibility of these active sites (Figure S26). Having mapped how the framework reorganizes during annealing, the next section turns from morphology and lattice evolution to the corresponding changes in metal valence states and local coordination environment, which control the electronic structure of the defect–metal interfaces.

### 2.2 X‐Ray Spectroscopy and Surface Characterization

To clarify how thermal annealing reorganizes the metal valence states and local coordination environment, X‐ray photoelectron spectroscopy (XPS), Ni K‐edge X‐ray absorption spectroscopy (XANES/EXAFS), and soft X‐ray absorption spectroscopy (sXAS) were combined.

High‐resolution Ni 2p XPS spectra (Figure 2a) show that PBA‐RT, PBA‐200 and PBA‐300 are dominated by oxidized Ni^2^^+^/Ni^3^^+^ components. In PBA‐350, these features weaken, and a distinct Ni^0^ contribution emerges, which becomes dominant in PBA‐450. Concomitantly, the Ni^2^^+^/Ni^3^^+^ peaks shift gradually to lower binding energy with increasing temperature, reflecting electronic modulation by CN vacancies and local coordination rearrangement [29]. For Fe, the 2p region is largely consistent with Fe^2^^+^ up to 350°C, whereas PBA‐450 displays additional Fe^0^ signatures, indicating partial reduction of the framework at the highest temperature. Mn 2p spectra retain mixed Mn^2^^+^/Mn^3^^+^ states with a slight shift to lower binding energy, while Co 2p spectra show Co^2^^+^/Co^3^^+^ at all temperatures up to 350°C, with a Co^0^ component appearing only in PBA‐450 (Figure S27) [30]. Taken together, these trends indicate that Ni is the most labile cation, undergoing partial reduction and exsolution at 350°C, whereas extensive Fe and Co reduction only occurs when the framework collapses at 450°C.

**FIGURE 2:**
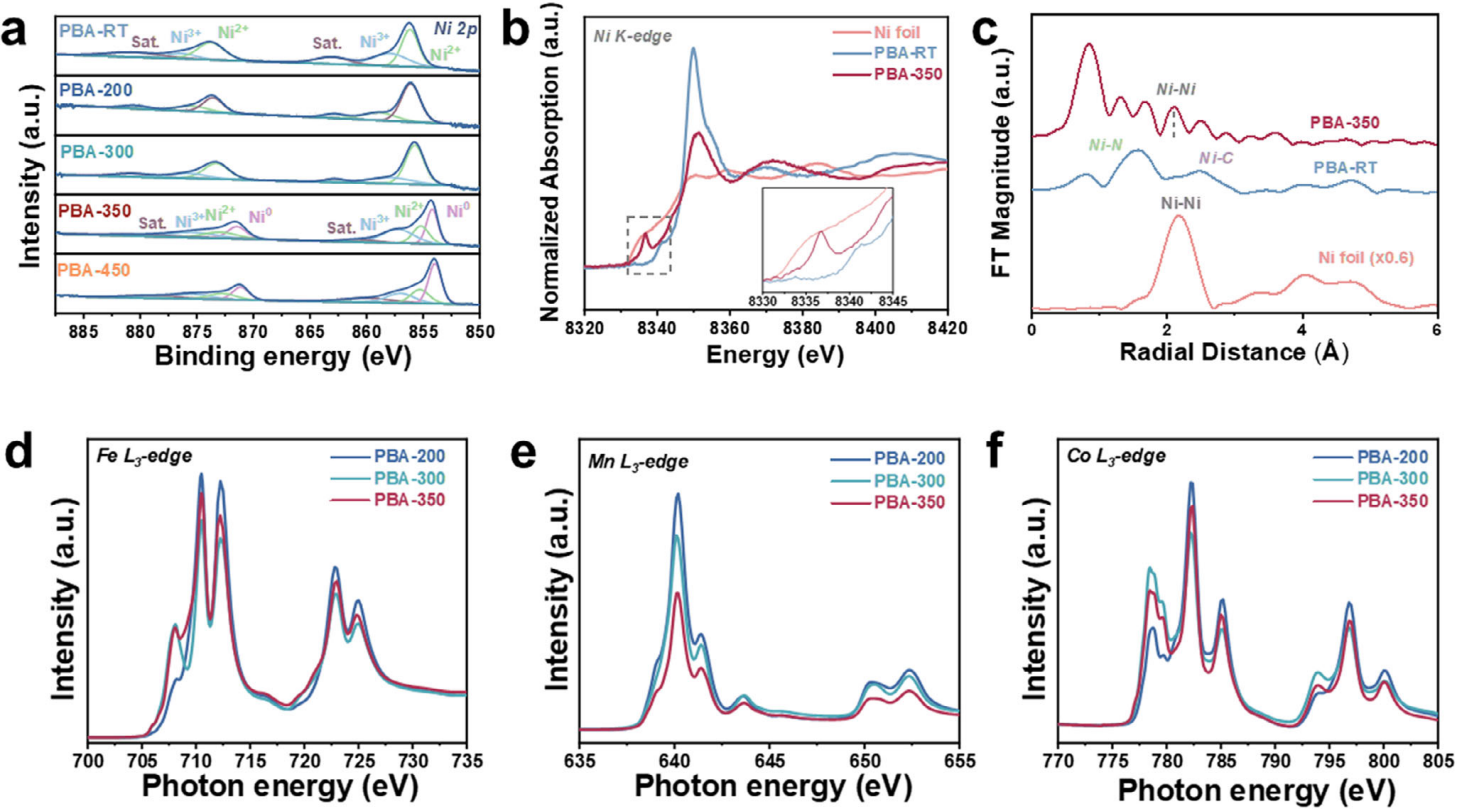
Electronic‐structure evolution and selective Ni reduction during thermal transformation. (a) High‐resolution Ni 2p XPS spectra of PBA‐RT, PBA‐200, PBA‐300, PBA‐350 and PBA‐450, showing the gradual shift from oxidized Ni^2^^+^/Ni^3^^+^ species to metallic Ni^0^ as temperature increases. (b) Ni K‐edge XANES spectra of PBA‐RT, PBA‐350 and Ni foil, highlighting the edge shift and reduced white‐line intensity in PBA‐350 characteristic of partial Ni reduction. (c) k^2^‐weighted Fourier transforms of the Ni K‐edge EXAFS spectra, evidencing the appearance of a Ni–Ni coordination shell in PBA‐350 alongside attenuated Ni–C/N contributions. (d–f) Fe L_3_‐, Mn L_3_‐ and Co L_3_‐edge soft X‐ray absorption spectra (sXAS) of PBA‐200, PBA‐300 and PBA‐350, showing subtle edge shifts and intensity changes consistent with vacancy‐driven electronic redistribution and selective reduction of 3d metals.

Bulk‐sensitive Ni K‐edge XANES further corroborates the emergence of metallic Ni in PBA‐350 (Figure 2b). The spectrum of PBA‐RT exhibits a higher edge energy and intense white line, characteristic of oxidized Ni in a cyanide‐ligated environment. By contrast, PBA‐350 displays a clear edge shift to lower energy and a reduced white‐line intensity, yielding a line shape that moves toward that of metallic Ni foil. These changes provide direct evidence for valence lowering and partial Ni^0^ formation within the PBA‐350 nanocages, whereas PBA‐RT remains fully oxidized.

The corresponding Fourier transforms of the EXAFS spectra in R‐space reveal how the local coordination environment reorganizes during annealing (Figure 2c). For PBA‐RT, the spectrum is dominated by Ni–C and Ni–N shells, confirming that Ni resides in a cyanide‐coordinated framework [31]. In PBA‐350, these Ni–C/N contributions are attenuated, and a pronounced Ni–Ni peak appears at ∼2.2–2.5 Å, demonstrating the formation of metallic Ni domains and disruption of the purely cyanide‐ligated environment. The Ni–Ni amplitude remains lower than in Ni foil, consistent with nanoscale metallic particles rather than bulk metal. An additional feature near 1.0 Å indicates residual Ni coordination and increased local disorder [32], reflecting the coexistence of metallic clusters and defect‐rich framework sites. Wavelet transform analysis of the EXAFS (Figure S28) confirms the appearance of high‐k, high‐R Ni–Ni scattering in PBA‐350, absent in PBA‐RT. Because PBA‐350 contains a heterogeneous Ni environment in which residual Ni‐C/N coordination, nanoscale Ni‐Ni domains, and vacancy‐rich framework sites coexist within the same particles, the Ni K‐edge EXAFS spectra are used to resolve reliable qualitative and semi‐quantitative coordination trends, not to extract absolute coordination numbers, Debye‐Waller factors, or vacancy concentrations through shell‐by‐shell fitting. Under such multimodal local environments, shell‐by‐shell fitting is sensitive to model assumptions and prone to overinterpretation. All structural assignments, namely attenuation of Ni‐C/N coordination, emergence of Ni‐Ni scattering and vacancy‐rich framework retention, are independently cross‐validated by XPS, sXAS, Raman, EPR, in situ SXRD and TEM/EDS. Particle‐size statistics derived from representative TEM and HAADF‐STEM images (Figures S12, S14, and S16) confirm that PBA‐350 contains finely dispersed Ni‐rich domains of approximately 10 nm, while higher‐temperature treatment produces particle coarsening and framework collapse.

Soft X‐ray absorption spectroscopy at the 3d metal L‐edges provides complementary surface‐sensitive insight into how multimetal redox chemistry responds to defect formation (Figure 2d; Figures S29 and S30) [33, 34]. The Fe L_3_‐edge spectra of PBA‐200, PBA‐300 and PBA‐350 display nearly unchanged edge positions but a gradual decrease in white‐line intensity, consistent with partial Fe^3+^ reduction and modest coordination relaxation. Mn L_3_‐edge spectra likewise show attenuated white lines, suggesting subtle changes in the Mn^2+^/Mn^3+^ balance. At the Co L‐edge, the low‐energy feature at ∼778–779 eV grows from PBA‐200 to PBA‐300 and slightly decreases in PBA‐350, while the main white line near ∼782 eV follows the opposite trend and shifts marginally to lower energy. This behavior points to partial Co reduction and ligand‐field weakening prior to extensive Co migration [35]. The Ni L_3_‐edge exhibits the strongest evolution: from PBA‐200 to PBA‐350, the white‐line intensity decreases, and the edge shifts to lower energy, confirming valence lowering and the appearance of a metallic Ni component in PBA‐350 in agreement with Ni 2p XPS and Ni K‐edge XANES/EXAFS.

Overall, these spectroscopic and microscopic results show that low‐temperature annealing selectively drives Ni exsolution and partial reduction while preserving a largely oxidized Fe/Mn/Co cyanide framework up to 350°C. The FeMn@CoNi core‐shell architecture is particularly well suited to this design strategy because it programs three properties simultaneously: (i) spatial programming, where the less thermally stable FeMn‐rich core decomposes first, directing internal hollowing inward while the more stable CoNi‐rich shell preserves the external nanocage morphology; (ii) selective exsolution chemistry, where Ni^2+^ in the CoNi shell is the most easily reduced cation in the system (confirmed by Ni 2p XPS, Ni K‐edge XANES/EXAFS and Ni L_3_‐edge sXAS in Figure 2), so thermal activation selectively releases Ni rather than Co, Fe, or Mn; and (iii) in situ defect‐metal coupling, where CN bond cleavage in the shell simultaneously generates CN vacancies and supplies the exsolved Ni, producing intrinsically electronically coupled Ni/PBA‐VCN interfaces rather than externally deposited Ni‐on‐PBA composites. The FeMn@CoNi core‐shell PBA is therefore not merely a multimetallic precursor, but a structural template that programs hollow cage formation, vacancy generation and Ni release into a single concerted reconstruction. The resulting PBA‐350 nanocages thus host metallic Ni nanoparticles electronically coupled to a defect‐rich multimetal lattice, establishing the cooperative defect–metal motifs that underpin the enhanced HER activity discussed in the following section.

### 2.3 HER Electrocatalytic Performance

We next evaluated how vacancy‐driven Ni exsolution affects alkaline HER activity. The HER performance of PBA‐RT, PBA‐200, PBA‐300, PBA‐350, and PBA‐450 was measured in 1.0 m KOH using a three‐electrode configuration, with commercial Pt/C supported on Ni foam as a benchmark. The contribution of the Ni foam substrate was rigorously assessed using control data from our previous work carried out under the identical Ni foam, catalyst loading, and alkaline HER protocol [6]. Bare Ni foam exhibits markedly inferior performance on every diagnostic metric: substantially larger overpotentials to reach equivalent current densities, a Tafel slope of 149.5 mV dec^−^^1^ compared with 56.4 mV dec^−^^1^ for PBA‐350, a much larger charge‐transfer resistance in EIS, and low ECSA‐normalized specific activity. Because substrate, loading, and electrolyte are identical across studies, this comparison is directly transferable and conclusively excludes the substrate as the dominant activity source. The Ni foam in the present measurements therefore functions strictly as a porous, conductive current collector. Linear sweep voltammetry (LSV) reveals that PBA‐350 is clearly the most active among the PBA series and approaches the behavior of Pt/C (Figure 3a). At low overpotentials, the current density of PBA‐350 rises steeply, whereas PBA‐RT, PBA‐200, PBA‐300, and PBA‐450 display more sluggish responses. The overpotentials required to reach −10 mA cm^−^^2^ (η_10_) follow the trend PBA‐350< PBA‐300 ≈ PBA‐200< PBA‐RT ≈ PBA‐450, with values of 28.4, 52.5, 54.1, 73.3, and 71.6 mV, respectively, compared with 23.3 mV for Pt/C (Figure 3b). This activity maximum at 350°C indicates that efficient HER requires the balanced formation of hollow nanocages, CN vacancies, and defect–metal interfaces, whereas excessive annealing at 450°C causes framework collapse and weakens the catalytic response.

**FIGURE 3:**
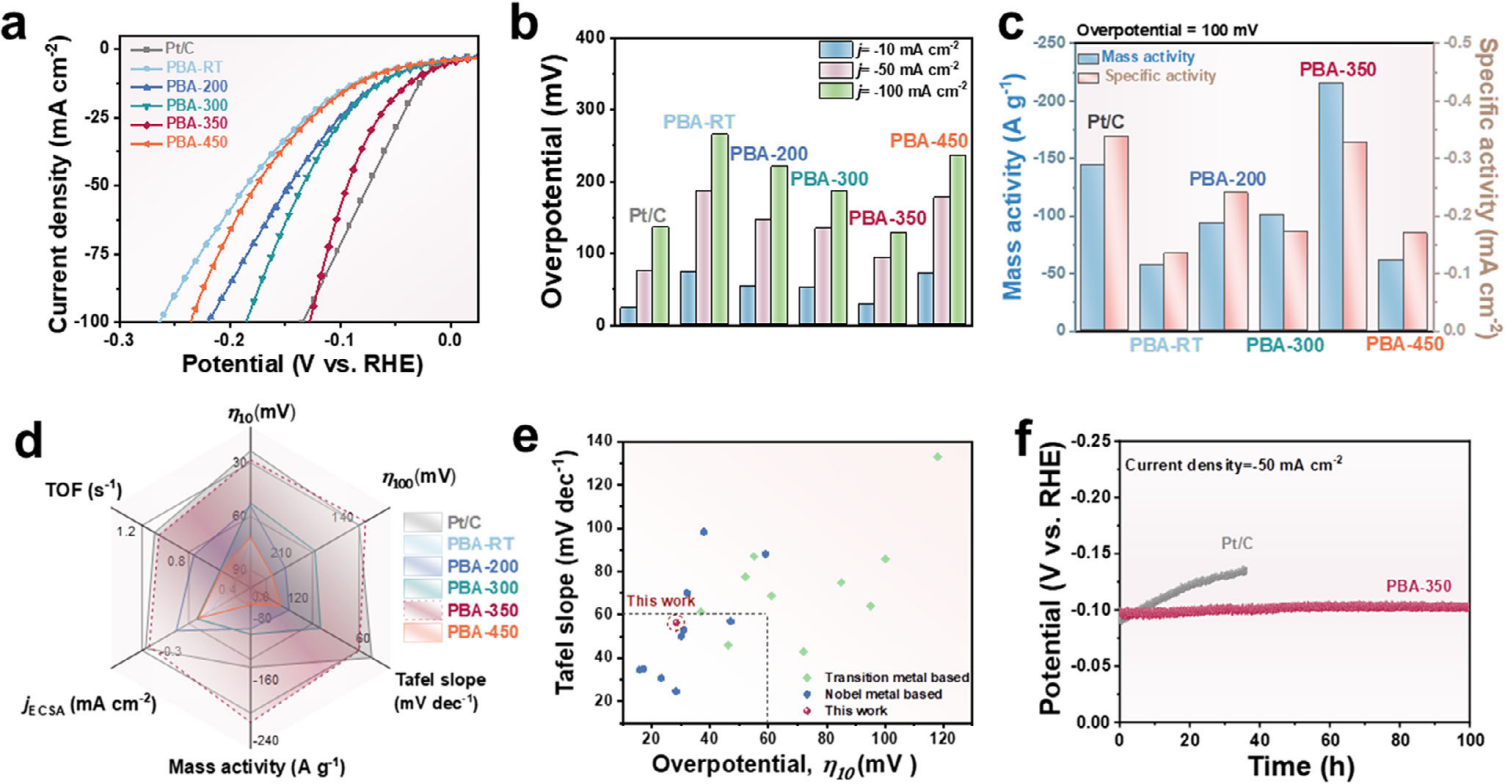
Defect–metal nanocages deliver Pt‐like HER activity in alkaline media. (a) Linear sweep voltammetry (LSV) curves for Pt/C, PBA‐RT, PBA‐200, PBA‐300, PBA‐350, and PBA‐450 in 1.0 m KOH, showing PBA‐350 approaching the activity of Pt/C and outperforming other PBA‐derived catalysts. (b) Overpotentials at −10, −50, and −100 mA cm^−^^2^ extracted from (a), highlighting the lowest η_10_ for PBA‐350. (c) Mass activity and ECSA‐normalized specific activity of the different catalysts, evidencing the intrinsically high activity of PBA‐350. (d) Radar plot comparing key HER performance indicators (η_10_, η_100_, Tafel slope, specific activity, mass activity, and TOF) for Pt/C and the PBA series, illustrating the balanced catalytic profile of PBA‐350. (e) Benchmarking of PBA‐350 against reported alkaline HER catalysts in terms of η_10_ and Tafel slope, demonstrating competitiveness with state‐of‐the‐art transition‐metal and noble‐metal systems. (f) Chronopotentiometry at −50 mA cm^−^^2^ over 100 h in 1.0 m KOH, showing negligible potential drift for PBA‐350.

To further evaluate the accessible active sites generated at different annealing temperatures, the geometric currents were normalized by the electrochemically active surface area (ECSA), obtained from double‐layer capacitance measurements (Figures S31 and S32) [36]. The ECSA values of the PBA series were compared as a quantitative descriptor of electrochemically accessible surface sites. PBA‐350 displays the largest ECSA (178 cm^2^) among the PBA series, indicating that it contains the largest population of electrochemically accessible sites. Using the surface site density adopted for the TOF analysis (Experimental Section, Supporting Information), these ECSA values correspond to approximately 2.88 × 10^−7^ mol (PBA‐RT), 2.62 × 10^−7^ mol (PBA‐200), 3.91 × 10^−7^ mol (PBA‐300), 4.43 × 10^−7^ mol (PBA‐350) and 2.45 × 10^−7^ mol (PBA‐450) of electrochemically accessible sites. The active‐site count follows a non‐monotonic temperature dependence, rising from PBA‐RT to PBA‐350 as hollow nanocage formation and Ni exsolution increase the electrochemically accessible surface, and then falling sharply in PBA‐450 as framework collapse reduces site accessibility. This trend directly parallels the HER activity series and confirms that 350°C represents the optimal reconstruction window. Critically, after ECSA normalization, PBA‐350 still exhibits the highest specific activity and TOF in the series, closely approaching those of Pt/C (Figure 3c; Figures S33 and S34), demonstrating that the overall activity enhancement reflects both the largest accessible‐site population and the fastest intrinsic kinetics per site rather than surface area alone. Importantly, this level of activity is achieved using only earth‐abundant metals.

Faradaic efficiency (FE) for H_2_ was also evaluated. Under the cathodic conditions applied here, potentials between 0 and −0.3 V (versus RHE) in 1.0 m KOH, H_2_ is the only thermodynamically and kinetically accessible cathodic product, and no competing side reactions occur in this window. The FE for H_2_ production by PBA‐350 is therefore intrinsically near unity, consistent with the stable chronopotentiometric response at −50 mA cm^−2^ over 100 h (Figure 3f), in which no anomalous current fluctuations or potential drift indicative of parasitic reactions are observed.

Kinetic analysis from Tafel plots provides further insight (Figure S35). PBA‐350 exhibits a Tafel slope of 56.4 mV dec^−^^1^, substantially lower than PBA‐RT (144.8 mV dec^−^^1^), PBA‐200 (116.7 mV dec^−^^1^), PBA‐300 (89.7 mV dec^−^^1^) and PBA‐450 (124.1 mV dec^−^^1^). This value is characteristic of a Volmer–Heyrovský mechanism in which electrochemical desorption is rate‐determining [37], and it signals that Ni exsolution and vacancy formation effectively accelerate both water dissociation and subsequent hydrogen evolution. Electrochemical impedance spectroscopy supports this conclusion: Nyquist plots collected at −0.1 V versus RHE show that PBA‐350 has the smallest charge‐transfer resistance (R_ct_) among the PBA catalysts, indicating faster interfacial charge transport at the electrolyte–catalyst interface (Figure S36) [38]. A comprehensive comparison of key performance indicators is provided in the radar chart (Figure 3d), which clearly underscores the superior catalytic profile of PBA‐350. Importantly, enhanced HER activity cannot be simply attributed to the presence of Ni species alone. The annealing‐temperature‐dependent samples derived from the same PBA precursor reveal that PBA‐350 possesses an optimized vacancy‐rich defect/metal interface and the largest ECSA, whereas excessive annealing does not further improve the activity. This trend indicates that the controlled generation of vacancies and defect/metal interfaces simultaneously plays a key role in accelerating alkaline HER kinetics.

When benchmarked against state‐of‐the‐art alkaline HER catalysts, PBA‐350 ranks among the best non‐precious systems reported to date. A comparison of η_10_ and Tafel slopes with recently published transition‐metal and noble‐metal catalysts (Figure 3e; Table S1) shows that PBA‐350 (28.4 mV, 56.4 mV dec^−^^1^) rivals or surpasses many Ru‐, Rh‐ and Co‐based materials and approaches the performance of leading Ru/RuO_2_ and Pt‐based catalysts, while avoiding critical metals. This highlights the effectiveness of coupling vacancy formation with Ni exsolution in PBAs as a design strategy for high‐performance alkaline HER electrocatalysts.

Long‐term stability was assessed by chronopotentiometry at −50 mA cm^−2^ in 1.0 m KOH for 100 h. The potential required to sustain this current density remains essentially constant over the entire test for PBA‐350 (Figure 3f), and post‐test LSV shows only a minimal shift compared with the initial curve and Pt/C (Figure S37), indicating preserved electrochemical kinetics. Post‐operation characterization over three additional independent metrics confirms that PBA‐350 retains its structural, compositional and electronic identity after 100 h of alkaline HER at −50 mA cm^−2^. Elemental analysis (Figure S38) shows preserved Fe/Mn/Co/Ni ratios and limited metal leaching. HAADF‐STEM and STEM‐EDS mapping (Figure S39) confirm retention of the hollow nanocage morphology and the Ni/PBA interfacial architecture. High‐resolution XPS of Fe 2p, Co 2p, Ni 2p, and Mn 2p (Figure S40) reveals only partial surface reoxidation and rehybridization, without destruction of the defect‐metal motif [39]. The combination of Pt‐like activity, low Tafel slope, and excellent durability under harsh alkaline conditions underscores the robustness of the defect–metal nanocages and motivates a deeper mechanistic analysis of how vacancy‐driven Ni exsolution tunes water dissociation and hydrogen adsorption, as discussed in the following section.

### 2.4 HER Mechanism Elucidation

To uncover why PBA‐350 approaches Pt‐like alkaline HER performance, we probed the elementary steps and associated electronic structure with operando spectroscopy and theory. In alkaline media, the Volmer step (water dissociation, H_2_O + e^−^ → H* + OH^−^) is typically rate limiting, followed by a Heyrovský step (H_2_O + H* + e^−^ → H_2_ + OH^−^) that is highly sensitive to hydrogen adsorption energetics [40].

Operando Raman spectroscopy directly tracks potential‐dependent water activation at the PBA‐350 surface (Figure 4a; Figure S41). In the 3100–3600 cm^−^^1^ region, bands assigned to interfacial, hydrogen‐bonded water gradually lose intensity as the potential is swept from open circuit to −60 mV versus RHE. This continuous attenuation signals progressive depletion of molecular H_2_O at the interface and the build‐up of dissociation products, consistent with increasingly facile Volmer kinetics under cathodic polarization. Combined with the reduced Tafel slope (56.4 mV dec^−^^1^) observed for PBA‐350, these data indicate that vacancy formation and Ni exsolution jointly lower the barrier for the initial water‐dissociation step [36].

**FIGURE 4:**
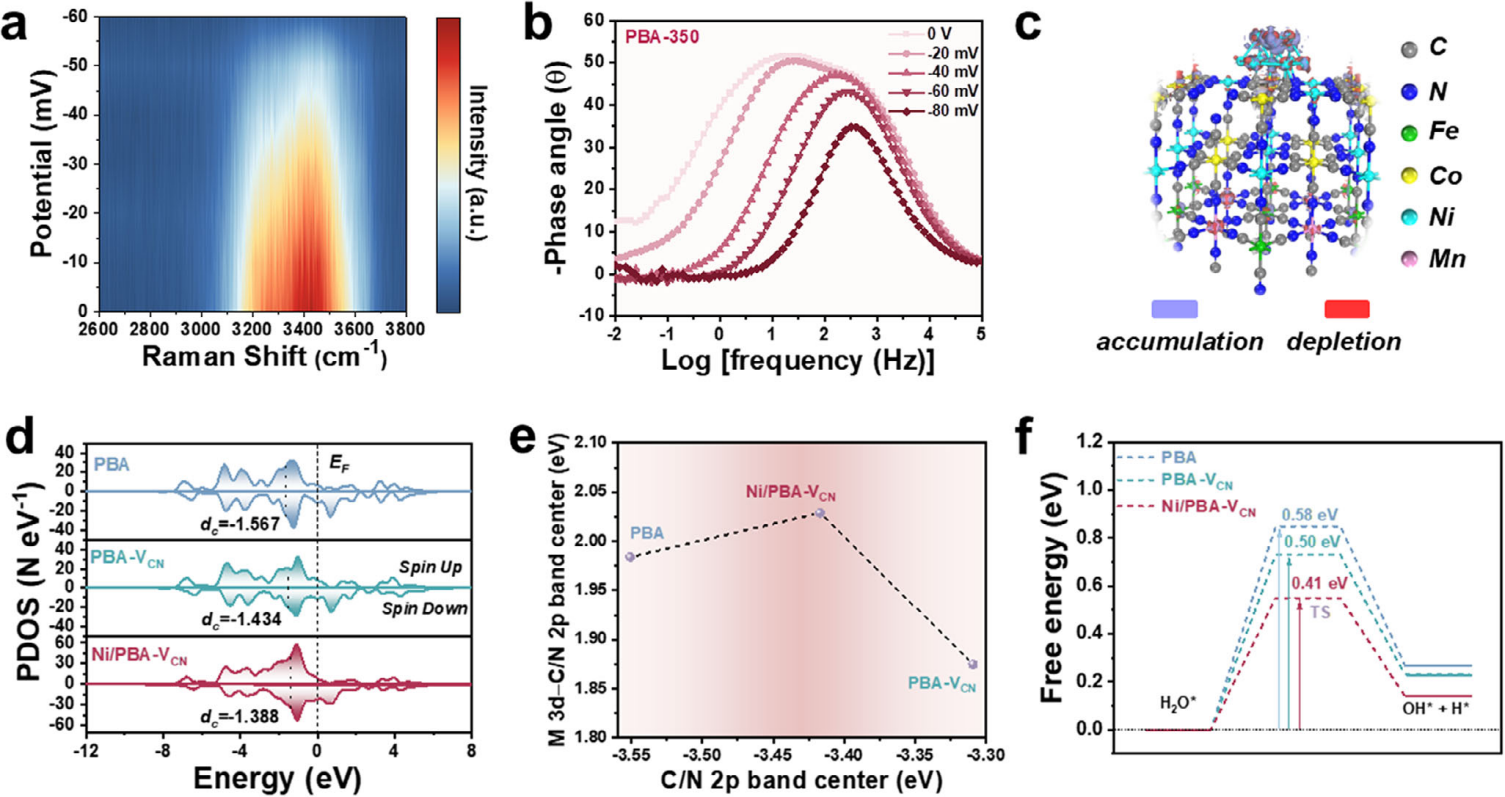
Dual‐site, vacancy‐assisted hydrogen evolution at Ni/PBA interfaces. (a) Operando Raman spectra of PBA‐350 recorded between 2600–3800 cm^−^^1^ as a function of applied potential, showing potential‐dependent changes in interfacial water structure and dissociation. (b) Bode phase plots for PBA‐350 at different potentials, evidencing enhanced interfacial charge‐transfer dynamics in PBA‐350 under HER conditions. (c) Atomic configuration of the Ni/PBA‐VCN model and corresponding charge‐density difference map, indicating strong electronic coupling between exsolved Ni and the vacancy‐rich PBA lattice. (d) Projected density of states (PDOS) of metal 3d orbitals for the three models, illustrating vacancy‐ and Ni‐induced tuning of the electronic structure near the Fermi level. (e) Energy separation between the metal 3d band center and the C/N 2p band center, used as a descriptor for metal–ligand covalency and correlated with HER activity. (f) Calculated activation barriers for water dissociation on PBA, PBA‐VCN and Ni/PBA‐VCN surfaces, identifying vacancy‐stabilized Ni sites as highly active Volmer centers.

To distinguish bulk from interfacial contributions to kinetics, operando EIS was performed for PBA‐RT and PBA‐350 (Figure 4b; Figure S42). In the Bode representation, the high‐frequency response, which associated with electronic transport within the catalyst, changes only weakly with potential for both samples, whereas the low‐frequency phase maximum, which reflects charge transfer across the electrolyte–catalyst interface, evolves strongly. For PBA‐350, this low‐frequency peak shifts and diminishes much more rapidly with increasing overpotential than for PBA‐RT, indicating a marked acceleration of interfacial charge transfer [41]. Phase‐angle analysis (Figure S43) quantifies this trend and shows shorter interfacial relaxation times for PBA‐350 across the entire potential range. Together with the operando Raman data, these results reveal that the hollow, vacancy‐rich framework decorated with exsolved Ni nanoparticles simultaneously accelerates water activation and electron delivery to adsorbate at the active interface.

Guided by these experimental insights, three slab models were constructed to disentangle the roles of vacancies and Ni exsolution: a pristine FeMn@CoNi PBA (PBA), a cyanide‐deficient framework (PBA‐V_CN_), and a Ni‐decorated, vacancy‐rich surface (Ni/PBA‐V_CN_), as shown in Figure S44. Charge‐density difference maps show pronounced electron redistribution at the Ni/PBA‐V_CN_ interface (Figure 4c; Figure S45), with electrons flowing from the Ni clusters and the FeMn core toward the CoNi shell. Plane‐averaged charge‐density profiles and work‐function calculations (Figure S46) reveal the formation of an internal electric field and a raised Fermi level upon Ni loading, which enhance the driving force for electron transfer to reaction intermediates [42, 43].

Electronic‐structure analysis links this charge redistribution to tuned metal–ligand covalency. Projected density of states (PDOS) show that PBA‐V_CN_ and Ni/PBA‐V_CN_ exhibit higher occupation near the Fermi level than pristine PBA, indicating enhanced conductivity (Figure S47) [44]. The metal d‐band center shifts from −1.567 eV (PBA) to −1.434 eV (PBA‐V_CN_) and −1.388 eV (Ni/PBA‐V_CN_), suggesting an enhanced overall electronic response and a stronger tendency for metal–adsorbate coupling after vacancy formation and Ni exsolution [45]. However, the active metal sites are embedded in a cyanide‐coordinated ligand framework, so that the averaged metal d‐band center alone cannot fully capture ligand‐mediated electronic regulation. Therefore, the energy separation Δ between the metal 3d band center and the C/N 2p band center was used as a complementary descriptor of M–CN covalency, reflecting the energetic alignment and hybridization between metal and ligand states. Similar to charge‐transfer energy descriptors in oxide electrocatalysts, a smaller Δ indicates stronger d–p hybridization and enhanced covalent interaction. CN vacancies raise the C/N 2p levels and decrease Δ, thereby strengthening M–CN hybridization and facilitating interfacial charge redistribution (Figure 4d,e; Figure S48). Furthermore, Ni loading partially re‐separates these states, yielding an intermediate Δ that balances electron donation and back‐donation to intermediates [46]. In other words, vacancy engineering “switches on” metal–ligand covalency, and Ni exsolution fine‐tunes it into the optimal window for HER. The Δ descriptor therefore provides information that is by construction inaccessible to the conventional metal‐only d‐band center: in PBA‐derived catalysts, HER kinetics are strongly modulated by metal‐ligand hybridization and interfacial charge transfer, and a smaller Δ captures these ligand‐mediated effects directly. Compared with the d‐band center alone, Δ more completely explains how Ni exsolution tunes the Ni/PBA‐VCN interface to simultaneously balance H_2_O activation, H* binding, and OH* removal.

We next quantified how these electronic changes impact the key Volmer step. Calculated H_2_O adsorption energies (E_ads_) show that Ni sites bind water more strongly than Co sites across all three models, and that Ni in Ni/PBA‐V_CN_ exhibits the largest E_ads_, reflecting the strongest interaction with oxygenated species (Figure S49). The O‐2p PDOS of adsorbed H_2_O on Ni sites in Ni/PBA‐V_CN_ shifts further away from the Fermi level (Figure S50), and 2D charge‐density difference maps reveal pronounced electron depletion/accumulation at the Ni–H_2_O interface, both signatures of strengthened bonding and efficient site‐to‐site electron transfer. Under these conditions, the computed transition‐state barrier for water dissociation on Ni/PBA‐V_CN_ is substantially lower than on PBA or PBA‐V_CN_ (Figure 4f). Thus, vacancy‐stabilized Ni sites act as highly active Volmer centers that cleave H_2_O with minimal energetic penalty. In parallel, Co sites on Ni/PBA‐V_CN_ display more favorable OH* desorption free energies than in the vacancy‐free model (Figure S51), preventing surface poisoning by strongly bound hydroxyls.

Finally, the Heyrovský step was analyzed through the hydrogen adsorption free energy, ΔG_H*_, on the three surfaces (Figure S52a). Both PBA‐V_CN_ and Ni/PBA‐V_CN_ exhibit significantly smaller |ΔG_H*_| than pristine PBA, indicating more balanced H* binding. The most striking site is located at the Ni/PBA‐V_CN_ interface, where ΔG_H*_ ≈ −0.144 eV closely approaches the thermoneutral window required for rapid H_2_ evolution. The averaged d‐band center in Figure 4d and the site‐specific ΔG_H_* in Figure S52 are complementary descriptors operating at different length scales, not contradictory predictions. The d‐band center is computed as an average over the multimetal Fe/Mn/Co/Ni framework and reflects the overall electronic activation of the lattice. The upshift in Ni/PBA‐V_CN_ indicates that the system is electronically poised for stronger adsorbate coupling. ΔG_H_* is governed by the local adsorption configuration, coordination environment and metal‐H bonding strength at the specific Ni/PBA‐V_CN_ interfacial site. At this site, CN vacancies and Ni exsolution redistribute the electron density and regulate the occupation of metal‐H bonding and antibonding states, with the result that local H binding is tuned to the near‐thermoneutral region despite the averaged d‐band upshift. The Crystal orbital Hamilton population (COHP) analysis in Figure S52b independently confirms this picture by placing the Ni/PBA‐V_CN_ site in the optimal metal‐H bonding window, evidencing an optimal metal–hydrogen bond strength that is neither too weak to activate H_2_O nor too strong to release H_2_. To further visualize this process, a mechanistic schematic was constructed for the Ni/PBA‐V_CN_ interface (Figure S52c). The cycle proceeds through: (i) the active site configuration at the Ni/PBA‐VCN interface, (ii) H_2_O adsorption, (iii) H_2_O dissociation at the vacancy‐stabilized Ni site with concomitant H* formation (Volmer step), (iv) *H_2_ formation through the Heyrovský step, and (v) H_2_ desorption, with OH* removal occurring concurrently at the adjacent Co site. The scheme makes explicit how vacancy‐stabilized Ni for water dissociation and adjacent Co for OH* removal collectively address the two kinetic bottlenecks of alkaline HER. Taken together, operando spectroscopy and theory identify PBA‐350 as the optimal state along a single reconstruction coordinate. Below 350°C, the cyanide framework is largely intact, but vacancy density and Ni exsolution are insufficient (PBA‐RT, PBA‐200, PBA‐300). Above 350°C, framework collapse, particle coarsening, and loss of cyanide coordination dominate (PBA‐450). At 350°C, three structural elements emerge concurrently: thin‐shell hollow nanocages (Figure 1b; Figures S12 and S13), a high density of CN vacancies (Figure 1d; Figure S22), and finely dispersed Ni domains coupled to the parent lattice (Figure 2b,c). Electrochemically, this combination produces both the largest ECSA in the series (178 cm^2^, Figures S31 and S32) and the highest TOF (Figure 3c; Figure S34), demonstrating that the enhancement is not merely surface area driven but reflects intrinsically faster kinetics per site. Mechanistically, operando Raman, EIS and DFT (Figure 4; Figure S52) show that vacancy‐stabilized Ni sites lower the Volmer barrier, adjacent Co sites accelerate OH* removal, and vacancy‐modified metal‐ligand covalency tunes H* binding toward thermoneutrality. The superior HER performance of PBA‐350 therefore originates from the cooperative coupling of hollow architecture, CN vacancies, selective Ni exsolution and dual‐site electronic modulation, rather than from any single structural or compositional factor, establishing vacancy‐assisted metal exsolution as a design principle for alkaline HER electrocatalysts. Whether this mechanistic advantage translates into device‐level performance under industrially relevant operating conditions is examined next in an anion‐exchange membrane water electrolyzer configuration.

### 2.5 AEM‐WE Device Performance

To assess whether the defect–metal architecture of PBA‐350 translates into device‐level performance, an AEMWE was assembled in a catalyst‐coated substrate configuration (Figure 5a). The membrane–electrode assembly comprised PBA‐350 deposited on Ti mesh as the anode and on carbon paper as the cathode, separated by a commercial PiperION membrane and operated in 1.0 m KOH. For comparison, a benchmark cell using commercial Pt/C (cathode) and IrO_2_ (anode) was tested under identical conditions.

**FIGURE 5:**
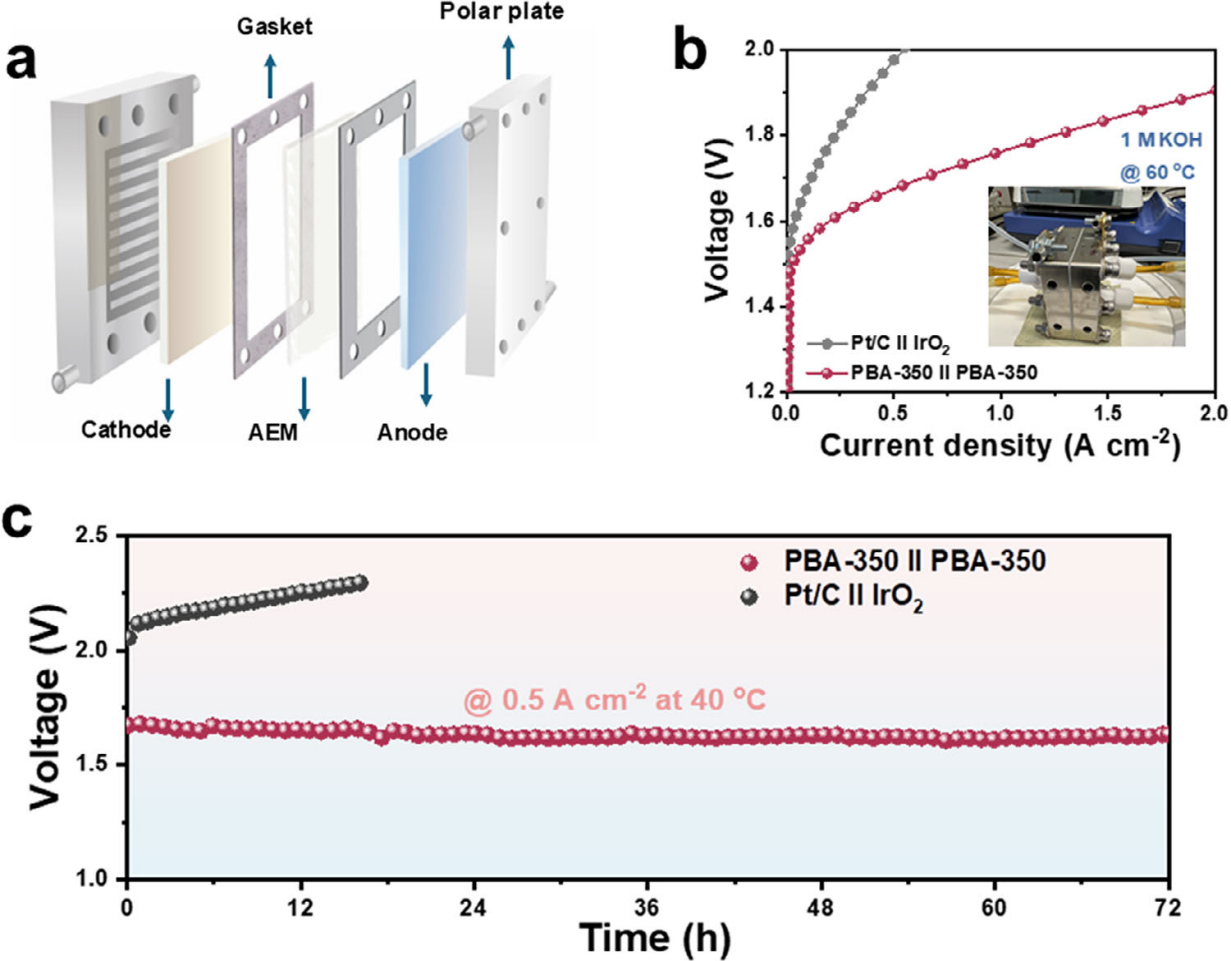
Integration of PBA‐350 into an anion‐exchange membrane water electrolyzer. (a) Schematic of the catalyst‐coated substrate (CCS) anion‐exchange membrane water electrolyzer (AEMWE) configuration, employing PBA‐350 on Ti mesh as the anode and PBA‐350 on carbon paper as the cathode, separated by a PiperION AEM. (b) Polarization curves of PBA‐350‖PBA‐350 and Pt/C‖IrO_2_ AEMWEs in 1.0 m KOH at 60°C, showing that the PBA‐350 cell reaches 1.0 A cm^−^^2^ at lower voltage than the noble‐metal benchmark. (c) Chronopotentiometry at 0.5 A cm^−^^2^ and 40°C for PBA‐350‖PBA‐350 and Pt/C‖IrO_2_ couples, demonstrating the superior voltage stability and durability of the PBA‐350‐based device under continuous operation.

The polarization curves demonstrate that the PBA‐350‖PBA‐350 AEMWE delivers excellent overall water‐splitting performance (Figure 5b). At 60°C, the PBA‐350 cell requires only 1.76 V to reach 1.0 A cm^−^^2^ in 1.0 m KOH, outperforming the Pt/C‖IrO_2_ benchmark across the entire current‐density range. The lower cell voltage reflects the high intrinsic HER activity of PBA‐350 in combination with its bifunctional alkaline activity, demonstrating that the defect–metal nanocages remain effective when integrated into a realistic membrane–electrode assembly. The OER performance and activity‐enhancement mechanism of PBA‐derived multimetallic catalysts in alkaline media have been established in our previous work [15, 16], and the present comparison is a full‐cell water‐splitting performance evaluation rather than a claim of OER half‐cell superiority over IrO_2_. The ability to achieve 1.0 A cm^−^^2^ at a low cell voltage further highlights the promise of PBA‐350 for high‐current‐density AEMWE operation.

Durability tests highlight the robustness of PBA‐350 under continuous operation. At 40°C and 0.5 A cm^−2^, the PBA‐350‖PBA‐350 cell sustains stable operation for at least 72 h with negligible voltage increase, whereas the Pt/C‖IrO_2_ cell exhibits a more pronounced rise in cell voltage over the same period (Figure 5c). This test was conducted in a full two‐electrode membrane–electrode assembly, providing device‐level evidence beyond conventional half‐cell evaluation. Post‐operation XPS and compositional analysis of the PBA‐350 cathode reveal that the overall metal distribution and oxidation states are largely retained, with only minor Co oxidation and limited leaching (Figures S53 and S54). These results show that the vacancy‐stabilized Ni/PBA framework is not only highly active in half‐cell HER tests but also compatible with industrially relevant AEMWE conditions, motivating further evaluation under more challenging saline environments. Extended stack‐level lifetime tests at higher current densities, where outcomes are governed by membrane hydration, gas management, local heating and cell engineering rather than intrinsic catalyst activity, are identified as the appropriate next step for industrial‐scale validation.

### 2.6 HER Performance in Alkaline Seawater

To evaluate saline‐electrolyte tolerance under controlled conditions, HER performance was assessed in alkaline simulated seawater (1.0 m KOH + 0.5 m NaCl). PBA‐350 supported on Ti mesh maintains high activity in this challenging medium (Figure 6a,b). The LSV curves show that PBA‐350 clearly outperforms PBA‐RT and bare Ti mesh and remains comparable to Pt/C across a wide current range. In particular, PBA‐350 requires an overpotential of only 159.5 mV to reach −100 mA cm^−^^2^, a value close to that achieved in pure 1.0 m KOH and essentially indistinguishable from the Pt/C benchmark (Figure 6b). These results demonstrate that the defect–metal nanocages preserve their favorable HER kinetics in the presence of high salt concentrations [47]. Simulated seawater was used as the alkaline saline electrolyte to ensure a controlled, reproducible comparison of intrinsic chloride tolerance across PBA‐RT, PBA‐350, and Pt/C. Natural seawater introduces site‐ and season‐dependent organic matter, microorganisms, suspended particles, trace metal ions, and Mg^2^^+^/Ca^2^^+^ salts, all of which contribute electrode fouling, precipitation, competitive adsorption, and additional mass‐transfer effects. Such matrix variability convolves catalyst‐intrinsic behavior with water‐matrix effects and is not reproducible across batches or sampling sites. A defined chloride‐containing alkaline electrolyte therefore isolates the intrinsic effect of chloride on the HER interface and enables meaningful benchmarking. Real‐seawater testing using site‐specific feedstock is identified as the appropriate next step toward field deployment [5].

**FIGURE 6:**
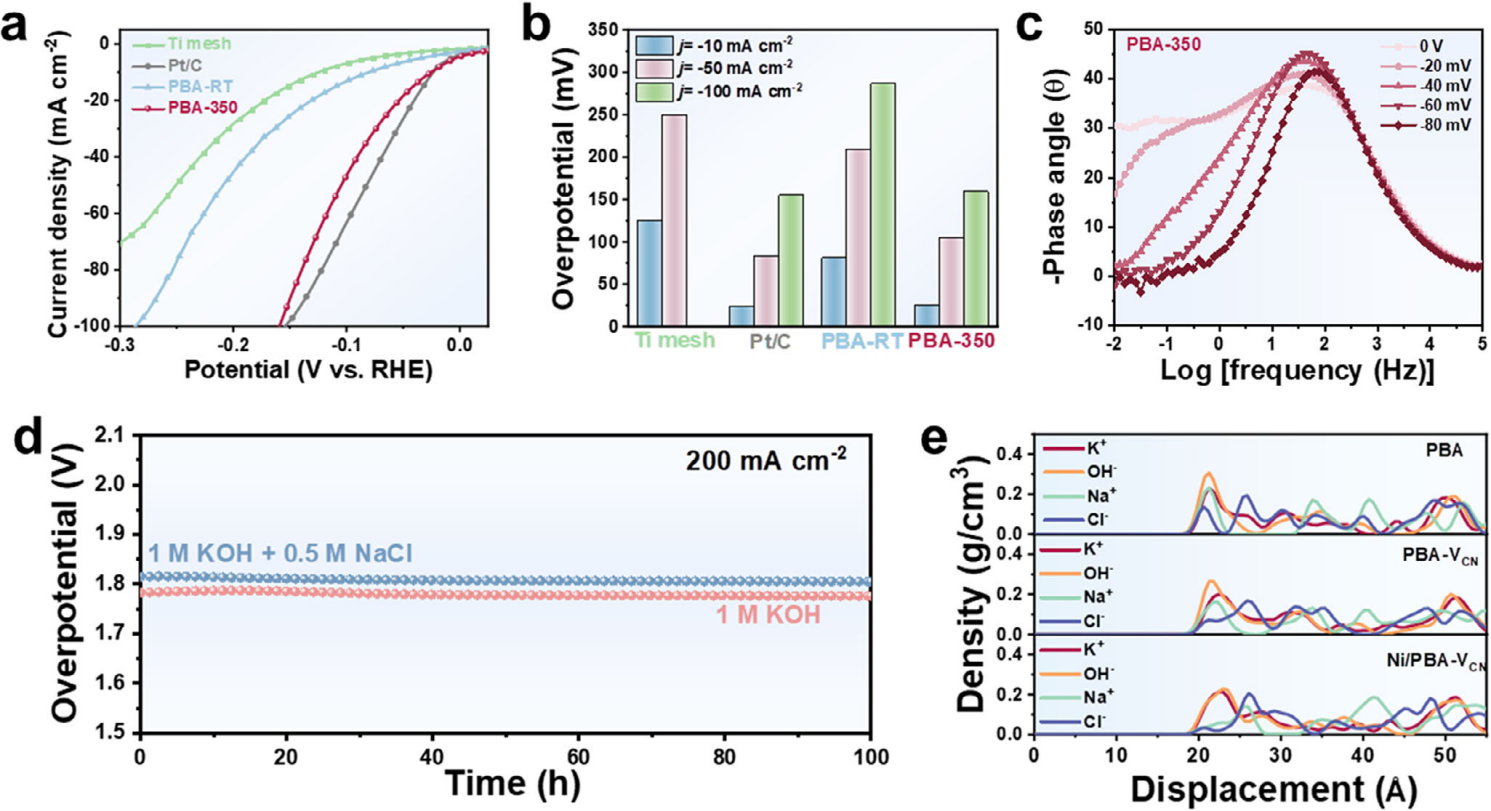
Robust hydrogen evolution and interfacial electrolyte structuring in simulated seawater. (a) LSV curves of PBA‐RT, PBA‐200, PBA‐300, PBA‐350, PBA‐450 and Pt/C in 1.0 m KOH + 0.5 m NaCl, showing that PBA‐350 maintains high activity in alkaline simulated seawater. (b) Overpotentials at −10, −50 and −100 mA cm^−^^2^ in simulated seawater, highlighting the low η required by PBA‐350. (c) Bode phase plots of PBA‐350 at different potentials in simulated seawater, showing accelerated interfacial charge transport under operating conditions. (d) Chronopotentiometric stability of a PBA‐350‖PBA‐350 two‐electrode cell at 200 mA cm^−^^2^ in 1.0 m KOH and in 1.0 m KOH + 0.5 m NaCl, evidencing long‐term durability in both freshwater and simulated seawater. (e) Ion‐density profiles above PBA, PBA‐VCN and Ni/PBA‐VCN surfaces obtained from molecular dynamics simulations, revealing salt‐ion exclusion near Ni/PBA‐VCN.

Kinetic parameters extracted from Tafel analysis and electrochemical impedance spectroscopy further support this conclusion. In 1.0 m KOH + 0.5 m NaCl, PBA‐350 exhibits a Tafel slope of 72.2 mV dec^−^^1^, markedly lower than that of PBA‐RT (151.9 mV dec^−^^1^) and Ti mesh (157.8 mV dec^−^^1^), indicating accelerated HER kinetics even under seawater conditions (Figure S55a). Nyquist plots collected at −0.1 V versus RHE show that PBA‐350 retains a much smaller charge‐transfer resistance than the reference electrodes (Figure S55b), confirming that fast interfacial charge transfer is preserved despite the presence of chloride and other ions [47]. To further probe the intrinsic HER activity under simulated seawater conditions, the TOF was estimated based on the ECSA‐derived accessible active sites (Table S2). PBA‐350 exhibits a higher TOF than PBA‐RT and approaches that of Pt/C, further confirming its accelerated intrinsic HER kinetics in seawater electrolyte. The FE for H_2_ in simulated seawater likewise remains near unity, as confirmed by the stable two‐electrode chronopotentiometric response at 200 mA cm^−2^ over 100 h (Figure 6d) with no evidence of competing cathodic processes. Operando EIS measurement was used to understand the improvement of the reaction kinetics in an alkaline seawater environment. As shown in Figure 6c and Figure S56, PBA‐350 presents boosted charge transfer at the catalyst/seawater electrolyte interface and improved tolerance in chloride‐containing alkaline electrolyte [48].

We then evaluated full‐cell stability in simulated seawater using a two‐electrode configuration with PBA‐350 as both cathode and anode (PBA‐350‖PBA‐350). This symmetric configuration was used to assess the integrated water‐splitting performance of PBA‐350 at the device level. At 200 mA cm^−2^, the cell shows only modest voltage drift over 100 h in 1.0 m KOH + 0.5 m NaCl (Figure 6d), comparable to its behavior in pure KOH and significantly more stable than a Pt/C‖IrO_2_ reference cell tested under the same conditions (Figure S57). These results demonstrate the practical full‐cell applicability of PBA‐350 in alkaline and chloride‐containing electrolytes, while the mechanistic focus of this work remains the vacancy‐assisted enhancement of alkaline HER. Post‐stability XPS and elemental analyses indicate that the recovered PBA‐350 electrode retains its surface chemical features and overall elemental distribution within the detection capability of the applied techniques (Figures S58 and S59), with no severe structural or compositional degradation observed. At the device level, the PBA‐350‖PBA‐350 cell maintains stable voltage over 100 h of continuous operation at 200 mA cm^−2^, while the Pt/C‖IrO_2_ reference cell shows more pronounced degradation under identical conditions. Together with the post‐mortem morphological and electronic stability evidence, these results establish the practical durability of PBA‐350 in chloride‐containing alkaline electrolyte. Quantitative ICP‐MS of the electrolyte and electrode‐resolved post‐mortem analysis are identified as valuable next steps for resolving trace metal dissolution and migration at the few‐ppm level.

To rationalize the unusual salt tolerance, molecular dynamics (MD) simulations of PBA, PBA‐VCN, and Ni/PBA‐VCN were performed in 1.0 m KOH + 0.5 m NaCl (Figure 6e; Figures S60 and S61). The ion density profiles show that Na^+^, K^+^, Cl^−^, and OH^−^ are displaced further from the Ni/PBA‐VCN surface than from PBA or PBA‐VCN, whereas water molecules preferentially accumulate within ∼5 Å of the Ni/PBA‐VCN interface. This results in a denser interfacial water layer with fewer competing ions at Ni/PBA‐VCN, effectively screening the active sites from corrosive species. The higher interfacial water density and reduced ion crowding correlate with the experimentally observed stability and low R_ct_ in seawater electrolyte [49].

Overall, the combination of half‐cell and two‐electrode tests, together with MD simulations, shows that vacancy‐stabilized Ni/PBA interfaces do not merely accelerate alkaline HER; they also sculpt the interfacial electrolyte structure, promoting a protective water layer that repels salt ions and contributes to the stable operation of PBA‐350 in simulated seawater.

## 3 Conclusions

Alkaline hydrogen evolution is constrained by slow water dissociation and by catalyst degradation in saline electrolytes. This work demonstrates that programming cyanide vacancies in multimetallic Prussian blue analogues triggers selective Ni exsolution and generates cooperative defect–metal interfaces that address both limitations. Controlled low‐temperature annealing of core–shell FeMn@CoNi PBAs converts solid nanocubes into hollow PBA‐350 nanocages, in which cyanide vacancies, internal voids and in situ exsolved Ni nanoparticles emerge in a single concerted reconstruction, producing a high density of strained defect–metal interfaces electronically coupled to a multimetal cyanide lattice.

Operando Raman, electrochemical impedance and X‐ray spectroscopy, combined with density functional theory, identify a dual‐site, defect‐assisted HER mechanism. Vacancy‐stabilized Ni sites at the Ni/PBA‐VCN interface lower the Volmer barrier and serve as the dominant water‐dissociation centers, while adjacent Co sites accelerate OH* removal and prevent surface poisoning. Vacancy‐modified metal–ligand covalency, captured by the energy separation between the metal 3d and C/N 2p band centers, tunes the hydrogen adsorption free energy toward thermoneutrality, enabling fast Heyrovský kinetics at the same interface. Molecular dynamics simulations further reveal a dense interfacial water layer at Ni/PBA‐VCN that displaces salt ions, rationalizing the exceptional chloride tolerance observed experimentally.

Translated into performance, PBA‐350 delivers an overpotential of 28.4 mV at 10 mA cm^−^^2^ and a Tafel slope of 56 mV dec^−^^1^ in 1.0 m KOH, approaching commercial Pt/C while relying solely on earth‐abundant metals, and shows negligible degradation over 100 h at −50 mA cm^−^^2^. Integration into an anion‐exchange membrane water electrolyzer delivers 1.76 V at 1.0 A cm^−^^2^ with stable operation at 0.5 A cm^−^^2^. In simulated alkaline seawater, PBA‐350 maintains comparable activity and stability at 200 mA cm^−^^2^, outperforming Pt/C‖IrO_2_ benchmarks under identical conditions. These device‐level results directly address the industrial requirements of low noble‐metal dependence, reduced energy loss, and stable operation at practically relevant current densities.

More broadly, vacancy‐assisted metal exsolution in multimetallic PBAs is established here as a general design principle for cooperative defect–metal electrocatalysts. By coupling programmable framework chemistry with controlled exsolution, the chemistry of multimetal coordination networks is converted into a single‐step route to robust, high‐activity architectures that bridge atomic‐scale interface engineering with device‐level performance. The two design levers demonstrated, namely vacancies that stabilize and electronically tune exsolved metals, and interfacial electrolyte sculpting that repels corrosive ions, are extendable beyond HER. Logical next targets include alkaline oxygen evolution, CO_2_ and nitrogen reduction, where similar dual‐site cooperative motifs are required, and where the framework‐programmable, atmosphere‐tunable exsolution chemistry shown here offers a versatile blueprint for designing next‐generation catalysts for scalable green hydrogen production and beyond.

## 4 Experimental Section

Experimental Procedures/Data are provided in the Supporting Information.

## Author Contributions

**Shiqi Wang**: conceptualization, investigation, writing – original draft, methodology. **Haixian Yan**: investigation, methodology. **Md Mofakkharulhashan**: methodology. **Hugo L. S. Santos**: investigation, methodology. **Wenyi Huo**: writing – review and editing, supervision, funding acquisition. **Pedro H. C. Camargo**: conceptualization, writing – review and editind, supervision, funding acquisition, resources, project administration.

## Conflicts of Interest

The authors declare no conflicts of interest.

## Supporting information




**Supporting File**: advs76307‐sup‐0001‐SuppMat.docx.

## Data Availability

The data that support the findings of this study are available from the corresponding author upon reasonable request.
