# Supplementary material for: Vacancy‐Driven Ni Exsolution in Prussian Blue Analogues Creates Cooperative Defect–Metal Sites for Alkaline Hydrogen Evolution
Source: Adv Sci (Weinh). 2026 Jul 3;13(53):e76307. doi: 10.1002/advs.76307 (PMC13334604; doi:10.1002/advs.76307)
Supplement: Supplementary file 1 [file ADVS-13-e76307-s001.docx]

**Supporting Information**

**Vacancy-Driven Ni Exsolution in Prussian Blue Analogues Creates Cooperative Defect–Metal Sites for Alkaline Hydrogen Evolution**

Shiqi Wang ^[a]*^, Haixian Yan ^[a]^, Hugo L. S. Santos ^[a]^, Md Mofakkharulhashan ^[a]^, Wenyi Huo ^[b]*^, and Pedro H.C. Camargo ^[a]*^

[a]	Dr. S. Wang, H. Yan, Dr. H. L. S. Santos, M. Mofakkharulhashan, Prof. P. H. C. Camargo
Department of Chemistry
University of Helsinki
A.I. Virtasen aukio 1, PO Box 55, Helsinki FIN–0014, Finland
E-mail: [pedro.camargo@helsinki.fi](mailto:pedro.camargo@helsinki.fi); shiqi.z.wang@helsinki.fi

[b]	Prof. W. Huo
NOMATEN Centre of Excellence
National Centre for Nuclear Research
Otwock 05-400, Poland

E-mail: wenyi.huo@ncbj.gov.pl

**Experimental Section**

**Materials**

All reagents were of analytical grade and used as supplied. Analytical grade Potassium hexacyanoferrate(III) (K_3_[Fe(CN)_6_], 99.9%, Sigma-Aldrich), Potassium hexacyanocobaltate(III) (K_3_[Co(CN)_6_], ≥99.0%, Sigma-Aldrich), Acetic acid nickel(II) salt (Ni(CH_3_CO_2_)_2_·4H_2_O, 98%, Sigma-Aldrich), Manganous acetate [Mn(CH_3_CO_2_)_2_·4H_2_O, ≥99.0%, Sigma-Aldrich], Sodium citrate dihydrate [HOC(COONa)(CH_2_COONa)_2_·4H_2_O, ≥99.0 %, Sigma-Aldrich], Sodium chloride (≥99.9%, Sigma-Aldrich), and Potassium hydroxide (KOH, Sigma-Aldrich) were applied without further purification. Deionized water with a resistivity of 18.2 MΩ∙cm was used in all experiments.

**Synthesis of core-shell PBA-RT nanocubes**

Core–shell FeMn@CoNi PBA nanocubes (PBA-RT) were synthesized following a modified coprecipitation procedure^16^. Typically, 0.3 mmol Ni(CH_3_CO_2_)_2_·4H_2_O, 0.4 mmol Mn(CH_3_CO_2_)_2_·4H_2_O, and 1mmol sodium citrate dihydrate (Na_3_C_6_H_5_O_7_·2H_2_O) were dissolved in 100 mL of deionized water and stirred for 30 min (solution A). 0.066 g K_3_[Fe(CN)_6_] and 0.066 g K_3_[Co(CN)_6_] were dissolved in 40 mL of deionized water with stirring for 5 min (solution B). Solution B was added dropwise to solution A and stirred for an additional 10 min. The mixture was aged for 24 h, and the resulting precipitate was collected by centrifugation, washed twice with deionized water, and dried under vacuum at 60 °C.

**Thermal treatment**

PBA-RT precursors were subjected to controlling annealing under Ar atmosphere to obtain PBA-200, PBA-300, PBA-350, and PBA-450. For a representative synthesis of PBA-200, 100 mg of PBA-RT was placed in a porcelain boat and heated in a tube furnace to 200 °C at 5 °C min^-1^, held for 150 min, and cooled naturally to room temperature. Analogous treatments at 300, 350, and 450 °C yielded PBA-300, PBA-350, and PBA-450, respectively.

**Off-line physical characterizations**

The morphology and composition of the samples were characterized by field emission scanning electron microscopy (FESEM, FEI Sirion, 20 kV) coupled with energy-dispersive X-ray spectroscopy (EDS). High-resolution transmission electron microscopy (HRTEM, Talos F200X) was employed to probe structural details. High-resolution synchrotron powder X-ray diffraction (PXRD, λ = 0.8272 Å) was collected at the MS/XPD beamline of SESAME (Allan, Jordan). X-ray photoelectron spectroscopy (XPS, Kratos Axis Ultra, Al Kα source) was calibrated with adventitious carbon (C 1s = 284.6 eV). Raman spectra were acquired with an Alpha 300 ACCESS spectrometer (532 nm excitation, 0.5 mW laser, 1200 mm^-1^ grating). Thermogravimetric analysis (TGA) was conducted under Ar up to 650 °C at 5 °C min^-1^. Inductively coupled plasma-mass spectrometry (ICP-MS) was used to determine metal ratios. Nitrogen adsorption-desorption isotherms were recorded at 77 K after degassing at 150 °C for 9 h, and surface areas were calculated by the Brunauer-Emmett-Teller (BET) method.

**Ex situ s-XAS measurements**

The X-ray absorption spectra of the Fe, Co, Mn and Ni L-edges were obtained in partial (PFY) fluorescence mode at the PIRX (Premiere InstRument for XAS, available photon energy from 100-2000 eV) beamline of SOLARIS National Synchrotron Radiation Centre, Poland. Catalyst samples were drop-cast onto conductive resin substrates.

**Operando synchrotron-based XRD measurement**

In situ synchrotron X-ray diffraction (XRD) in transmission mode was performed at the ID09-MS/XPD beamline of SESAME. Measurements were conducted with an incident wavelength of 0.8272 Å (25 keV). Samples were sealed in quartz capillaries under vacuum, and the transmitted X-rays produced diffraction rings that were collected by a two-dimensional CCD detector.

**XANES and EXAFS measurements**

X-ray absorption spectroscopy at the Ni K-edge was carried out in transmission mode using a RapidXAFS 2M spectrometer (Anhui Absorption Spectroscopy Analysis Instrument Co., Ltd.). Measurements employed Si(531) and Si(533) spherically bent crystal analyzers with a curvature radius of 500 mm. Reference spectra were recorded under identical conditions. The acquired data were normalized and analyzed using the ATHENA software package.

**Operando Raman measurement**

Operando Raman spectroscopy of PBA-350 during electrochemical operation was performed using an Alpha 300 ACCESS spectrometer (WITec) equipped with a 50× objective (Leica Microsystems). Each spectrum was collected from 20 successive scans with an exposure time of 5 s per scan and a resolution of ~1 cm^-1^. Raman signals were recorded at open-circuit potential and under applied potentials from 0 to −0.06 V versus RHE.

**Electrochemical test**

Electrochemical measurements were carried out at room temperature using a CHI 660E workstation (Shanghai Chenhua, China) and an Autolab PGSTAT302N potentiostat in a three-electrode configuration. Nickel foam (1×1 cm^2^) coated with catalyst served as the working electrode, a graphite rod as the counter electrode, and a Hg/HgO electrode (+0.098 V vs NHE at 25 °C) as the reference. Catalyst inks were prepared by dispersing 3 mg of sample in 1 mL of a Nafion-ethanol-water mixture (500 μL ethanol, 480 μL water, 20 μL Nafion, 5 wt%) using ultrasonic treatment in an ice bath for 1 h. An aliquot of 90 μL of the suspension was drop-cast onto Ni foam, resulting in a catalyst loading of ~0.27 mg cm^-2^, followed by drying at room temperature.

Electrochemical testing was conducted in 1 M KOH or 1 M KOH + 0.5 M NaCl electrolytes. The uncompensated resistance *R_s_* was determined from the high-frequency intercept of EIS (and by current-interrupt where indicated). During data acquisition, 85% dynamic *iR* compensation (positive feedback) was applied using the measured *R_s_*. Unless otherwise stated, all potentials and overpotentials are reported with 85% *iR* compensation applied. Linear sweep voltammetry (LSV) was recorded at 5 mV s^-1^, and all potentials were converted to the reversible hydrogen electrode (RHE) scale using E (vs RHE) = E (vs. Hg/HgO) + 0.0591pH + 0.098. For HER, the overpotential was defined as *η* = -E_RHE_ (referenced to 0 V vs RHE) and reported as a positive magnitude at the specified cathodic current densities. For overall water splitting in two-electrode measurements, the cell voltage *E_cell_* is reported without *iR* correction; where stated, the cell overpotential was calculated as *η*_cell_ **=** *E_cell_* – 1.23 V (the thermodynamic reversible voltage at 25 °C).

The electrochemically active surface area (ECSA) was estimated from double-layer capacitance values determined by cyclic voltammetry (20-100 mV s^-1^) in the non-Faradaic region, assuming a specific capacitance of 40 μF cm^-2^. Operando electrochemical impedance spectroscopy (EIS) was performed from 100 kHz to 0.1 Hz with a 10 mV AC perturbation under applied potentials of 0 to -0.08 V versus RHE. The turnover frequency (TOF) values were calculated according to the following equation: TOF = |j| × A / 2Fn, where j is the current density at a given overpotential (0.1 V in this work), A is the geometric electrode area, F is the Faraday constant, n is the amount of electrochemically accessible active sites, and the factor of 2 accounts for the two-electron transfer process required for generating one H₂ molecule. The number of active sites was estimated by ECSA. Specifically, the number of accessible surface metal sites was estimated according to: n = ECSA × Γ where n is the amount of accessible active sites, and Γ is the surface site density of metal atoms, taken as 2.49 × 10^-9^ mol cm^-2^, corresponding to approximately 1.5 × 10^15^ sites cm^-2^. Stability was assessed by chronopotentiometry at -50 mA cm^-2^ for 100 h in HER tests. Overall water splitting was evaluated in 1 M KOH using two PBA-350/NF electrodes (1 × 1 cm^2^) in a two-electrode configuration, operated at 200 mA cm^−2^ for 200 h. Unless stated otherwise, electrochemical data are representative of at least three independently prepared electrodes per catalyst. Key metrics, Tafel slopes and stability trends were reproduced across independent preparations.

**Anion Exchange Membrane Water Electrolyzers (AEMWE) fabrication**

Anion exchange membrane water electrolyzers (AEMWEs) were assembled without hot pressing by placing the cathode and anode on opposite sides of a PiperIon A60-HC03 membrane (60 μm thickness). Cathodes were prepared using either PBA-350 supported on carbon paper (2.2 × 2.2 cm^2^, 1.5 mg cm^−2^) or commercial Pt/C on carbon paper of identical size and loading. Anodes were prepared using PBA-350 deposited on Ti mesh or commercial IrO_2_/Ti mesh with the same geometric area and loading. The assembled cells were purchased from Suzhou Sinero Technology Co. Ltd. Electrolyzer performance was tested at 60 °C within a voltage range of 1.2-2.0 V, using 10 mV step intervals with 1 s per step. Durability was assessed at 40 °C and 500 mA cm^-2^ under continuous operation.

**Theoretical calculations**

Density functional theory (DFT) calculations were performed using the CASTEP module in Materials Studio package. The generalized gradient approximation with the Perdew-Burke-Ernzerhof (PBE)+U functional was employed, with on-site Coulomb corrections of U_Fe_ = 3.5 eV, U_Co_ = 3.5 eV, U_Ni_ = 3.0 eV, and U_Mn_ = 4.0 eV, according to previously reported values for transition-metal cyanide/PBA-derived systems and related transition-metal oxides/hydroxides (Advanced Materials, 2023, 35, 2304494). A plane-wave cutoff energy of 420 eV was applied together with ultrasoft pseudopotentials. The interactions between valence electrons and ionic cores were described using the OTFG ultrasoft pseudopotential method. Spin-polarized calculations were performed for all transition-metal-containing models to account for the magnetic nature of Fe, Co, Ni, and Mn sites. Structural relaxations were carried out using the limited-memory Broyden–Fletcher–Goldfarb–Shannon algorithm. Considering the large slab/supercell size, the Brillouin zone was sampled using a Γ-centered 1 × 1 × 1 Monkhorst–Pack k-point mesh. The convergence thresholds were set to 5 × 10^-5^ eV/atom for total energy, 0.001 eV/Å for maximum force, and 0.005 Å for maximum displacement. A vacuum spacing of 20 Å was introduced along the z-axis to avoid periodic interactions.

Surface slab models were constructed to represent the exposed PBA-derived catalytic surface. The CN-vacancy model was constructed by removing CN ligands from the surface supercell. Ni-containing surface models were further constructed by introducing Ni species around the vacancy sites to describe the reconstructed Ni-enriched active environment. The rationality of these models is supported by the experimental observations of hollow vacancy-rich PBA-derived structures and Ni-enriched/reconstructed surface species.

The HER activity was evaluated using the computational hydrogen electrode model. The water adsorption energy was calculated as Δ𝐸_𝐻2𝑂_ = 𝐸_𝑠𝑢𝑟𝑓+_ *_H_*_2O_ − 𝐸_𝑠𝑢𝑟𝑓_ − 𝐸 *_H_*_2O_, where 𝐸_𝑠𝑢𝑟𝑓_ and 𝐸_𝑠𝑢𝑟𝑓+𝐻2𝑂_ are the total energies of the surface before and after water adsorption, respectively, and 𝐸_𝐻2𝑂_ is the energy of a free water molecule. The Gibbs free energy of hydrogen adsorption was calculated as Δ𝐺_𝐻∗_ = Δ𝐸_𝐻∗_ + Δ𝑍𝑃𝐸 – 𝑇Δ𝑆, where Δ𝐸_𝐻∗_, Δ𝑍𝑃𝐸, 𝑇 and Δ𝑆 represent the binding energy, zero-point energy change, temperature, and entropy change of the H* adsorption system, respectively. Considering the negligible vibrational entropy of adsorbed H*, Δ𝑆 = 𝑆_𝐻_^∗^ −1/2 𝑆_𝐻2_ ≈ −1/2*S_H_*_2,_ and Δ𝑍𝑃𝐸 =𝑍𝑃𝐸_𝐻_^∗^ −1/2𝑍𝑃𝐸*_H_*_2_, therefore, the free energy of the adsorbed state can be calculated as Δ𝐺_𝐻_^∗^ = Δ𝐸_𝐻_^∗^ + 0.24 𝑒𝑉.

Molecular dynamics (MD) simulations were carried out to investigate the distribution of ions at the catalyst/electrolyte interface. The periodic boundary conditions were applied in the horizontal plane. A few layers of PBA, PBA-V_CN_, or Ni/PBA-V_CN_ were fixed at the bottom of the simulation box to represent the electrode surface. To simulate the electrolyte in the experiment (1 M KOH + 0.5 M NaCl), the solvent model contains 1000 H_2_O molecules, 20 Na^+^, 20 Cl^−^, 40 K^+^ and 40 OH^−^. The size of model box containing is about 131.96 Å × 122.73 Å ×127.50 Å. All calculations were performed at 300 K under the NVT ensemble for 3 ns to achieve equilibrium.

**Supplementary Figures**

**Figure S1**. **Thermal decomposition and exsolution window of the PBA precursor.** Thermogravimetric analysis (TGA) of the PBA‑RT precursor under Ar, showing dehydration below ~150 °C, onset of framework decomposition near ~200 °C, and extensive structural collapse above ~450 °C, coincident with accelerated exsolution/aggregation of metallic nanoparticles.

**Figure S2. Temperature-dependent morphology and elemental redistribution.** SEM images of (A) PBA‑RT, (B) PBA‑200, (C) PBA‑300, (D) PBA‑350 and (E) PBA‑450 with corresponding EDS profiles, tracking the evolution from solid nanocubes to hollow nanocages (350 °C) and, at higher temperature, framework collapse accompanied by metal segregation.

**Figure S3. High-resolution imaging of the defect–metal nanocages. (**A) High-resolution SEM image and (B) STEM image of PBA‑350, highlighting the preserved cubic morphology and thin-shell hollow architecture formed in the vacancy–exsolution regime.

**Figure S4. Optical response during thermal transformation.** UV/Vis diffuse-reflectance spectra of PBA‑RT, PBA‑200, PBA‑300, PBA‑350 and PBA‑450, showing temperature-dependent changes consistent with framework reconstruction and increasing metallic character.

**Figure S5**. **Pristine PBA microstructure.** (A,B) TEM images and (C) corresponding selected-area electron diffraction (SAED) pattern of PBA‑RT, confirming an ordered, polycrystalline cyanide framework prior to vacancy formation and Ni exsolution.

**Figure S6.** **Core–shell elemental architecture of PBA‑RT**. HAADF‑STEM images and STEM‑EDS elemental maps for PBA‑RT, revealing a Fe/Mn-enriched core and a Co/Ni-enriched shell that later governs hollowing and selective Ni exsolution.

**Figure S7****. Elemental line-scan across a PBA‑RT nanocube.** HAADF‑STEM image and corresponding EDS line-scan verifying spatial segregation of Fe/Mn (core) and Co/Ni (shell), providing the compositional scaffold for defect generation and exsolution upon annealing.

**Figure S8****. Early-stage hollowing and strain development at 200 °C.** (A) TEM image, (B) SAED pattern, (C) HRTEM image, (D) GPA strain contour map (εxy) and (E) EDS result of PBA‑200, showing the onset of internal void formation and local lattice distortion preceding Ni exsolution.

**Figure S9****. Elemental mapping of PBA‑200.** HAADF‑STEM images and STEM‑EDS elemental maps for PBA‑200, indicating preserved outer morphology with emerging internal reconstruction.

**Figure S10****. Progressive framework reconstruction at 300 °C.** (A) TEM image, (B) SAED pattern, (C) HRTEM image, (D) GPA strain contour map (εxy) and (E) EDS result of PBA‑300, revealing expanded cavities and increased strain as vacancies accumulate and the shell reorganises.

**Figure S11****. Elemental mapping of PBA‑300.** HAADF‑STEM images and STEM‑EDS elemental maps for PBA‑300, showing continued hollowing and incipient metal segregation in the shell.

**Figure S12.** **Formation of hollow nanocages with exsolved metal domains at 350 °C.** (A) TEM image, (B) SAED pattern, (C,D) HRTEM images and (E) EDS result of PBA‑350, evidencing a thin-shell hollow structure decorated with nanoscale Ni-rich particles (Ni-rich domains with an apparent average size of 9.7 ± 2.8 nm).

**Figure S13.** **Elemental mapping of PBA‑350 nanocages.** HAADF‑STEM images and STEM‑EDS elemental maps for PBA‑350, confirming uniform decoration of the nanocage shell by Ni-rich domains coupled to a defect-rich PBA framework.

**Figure S14. High-temperature exsolution and framework collapse at 450 °C.** (A) TEM image, (B) SAED pattern, (C) HRTEM image, (D) GPA strain contour map (εxy) and (E) EDS result of PBA‑450, showing coarsened metallic particles (17.7 ± 6.7 nm) and loss of framework integrity beyond the optimal vacancy–exsolution window.

**Figure S15.** **Elemental mapping of PBA‑450.** HAADF‑STEM images and STEM‑EDS elemental maps for PBA‑450, highlighting extensive metal segregation and diminished framework order.

**Figure S16****. Complete transformation at 700 °C.** (A) TEM image, (B) SAED pattern, (C) HRTEM image, (D) HAADF‑STEM images with STEM‑EDS maps and (E) EDS result of PBA‑700, showing full framework destruction and the formation of multimetallic alloy nanoparticles supported on N-doped carbon with an apparent size of 15.2 ± 5.5 nm.

**Figure S17****. Raman tracking of cyanide coordination and vacancy formation.** Temperature-dependent Raman spectra (room temperature to 550 °C) in the CN-stretching region, revealing the evolution of Fe–CN–Mn and Co–CN–Ni linkages and progressive CN vacancy generation during annealing.

**Figure S18.** **Low-frequency Raman signatures of framework distortion.** Temperature-dependent Raman spectra (room temperature to 550 °C; 100–400 cm⁻¹), capturing changes in metal–ligand vibrational modes associated with lattice softening, defect formation and reconstruction.

**Figure S19.** **In situ SXRD during thermal transformation.** In situ synchrotron XRD patterns collected upon heating PBA‑RT from room temperature to 500 °C, showing lattice expansion, peak broadening and the emergence of Ni (111), consistent with vacancy-assisted Ni exsolution.

**Figure S20.** **Ex situ SXRD across the annealing series.** Ex situ synchrotron XRD patterns of PBA‑RT, PBA‑200, PBA‑300, PBA‑350 and PBA‑450, tracking the transition from an ordered PBA lattice to a defect-rich nanocage phase and, at higher temperature, a Ni-rich collapsed phase.

**Figure S21.** **Temperature-dependent elemental composition.** Atomic fractions of Fe, Mn, Co and Ni in PBA-derived samples as a function of annealing temperature, highlighting preferential Ni exsolution and increasing Co depletion at elevated temperature.

**Figure S22.** **EPR evidence for vacancy-associated paramagnetic centres.** Electron paramagnetic resonance spectra of PBA‑RT, PBA‑200 and PBA‑350, showing the growth of a vacancy-linked signal that accompanies framework softening and Ni release.

**Figure S23.** **Multimetal composition controls hollowing and exsolution: FeCoMn-PBA.** HAADF‑STEM images and STEM‑EDS elemental maps for FeCoMn‑PBA annealed at 350 °C under Ar, providing a compositional control for cavity formation and defect generation.

**Figure S24.** **Multimetal composition controls hollowing and exsolution: FeCoNi-PBA.** HAADF‑STEM images and STEM‑EDS elemental maps for FeCoNi‑PBA annealed at 350 °C under Ar, showing temperature-driven reconstruction and metal redistribution distinct from the quaternary FeMn@CoNi system.

**Figure S25.** **Multimetal composition controls hollowing and exsolution: FeNiMn-PBA.** HAADF‑STEM images and STEM‑EDS elemental maps for FeNiMn‑PBA annealed at 350 °C under Ar, underscoring the role of Ni-containing domains in driving exsolution.

**Figure S26.** **Porosity increase upon nanocage formation.** Nitrogen adsorption–desorption isotherms of PBA‑RT and PBA‑350, confirming increased surface area and mesoporosity upon vacancy-driven hollowing and Ni exsolution.

.

**Figure S27.** **Surface oxidation states from XPS.** High-resolution XPS spectra of (A) Fe 2p, (B) Mn 2p, and (C) Co 2p for PBA‑RT, PBA‑200, PBA‑300, PBA‑350 and PBA‑450, revealing temperature-dependent electronic redistribution accompanying vacancy formation and exsolution.

**Figure S28.** **Wavelet-transform EXAFS confirms metallic Ni domains.** Wavelet transforms of k²-weighted Ni K-edge EXAFS for Ni foil, PBA‑RT and PBA‑350, resolving the emergence of Ni–Ni scattering in PBA‑350 and confirming nanoscale metallic Ni exsolution.

**Figure S29.** **Surface-sensitive sXAS of 3d metals.** Enlarged (A) Fe L₃-, (B) Mn L₃-, (C) Co L₃- and (D) Ni L₃-edge sXAS spectra of PBA‑200, PBA‑300 and PBA‑350, showing selective Ni valence lowering and coupled redox tuning of Fe/Mn/Co in the vacancy–exsolution regime.

**Figure S30. Ni L₃-edge evolution with annealing temperature.** Ni L₃-edge sXAS spectra of PBA‑200, PBA‑300 and PBA‑350, highlighting white-line suppression and edge shifts consistent with partial Ni⁰ character.

**Figure S31.** **CVs used to estimate double-layer capacitance.** Cyclic voltammograms for (A) Pt/C, (B) PBA‑RT, (C) PBA‑200, (D) PBA‑300, (E) PBA‑350 and (F) PBA‑450 in the non-faradaic region, used to determine Cdl and ECSA.

**Figure S32.** **Cdl and ECSA extraction for the catalyst series.** Derived Cdl values for (A) Pt/C, (B) PBA‑RT, (C) PBA‑200, (D) PBA‑300, (E) PBA‑350 and (F) PBA‑450. ECSA was calculated as ECSA = Cdl/(Cs·A), where Cs = 0.04 mF cm⁻² (alkaline media) and A = 1 cm². Calculated ECSAs are 212, 115.5, 105.3, 157, 178 and 98.3 cm², respectively.

**Figure S33. ECSA-normalised HER polarisation curves.** LSV curves from Fig. 3A normalised by ECSA, illustrating that PBA‑350 maintains superior intrinsic HER activity beyond surface-area effects.

**Figure S34.** **Turnover frequency (TOF) analysis.** TOF values for Pt/C and the PBA-derived catalysts as a function of overpotential, confirming accelerated intrinsic kinetics for PBA‑350.

**Figure S35.** **Tafel analysis of HER kinetics.** Tafel plots for Pt/C and the PBA series in 1.0 M KOH, showing the reduced slope of PBA‑350 consistent with faster Volmer–Heyrovský kinetics.

**Figure S36. Impedance response under HER conditions.** Nyquist plots recorded at −0.1 V versus RHE, evidencing a markedly lower charge-transfer resistance for PBA‑350 relative to other PBAs.

**Figure S37.** **Activity retention after long-term alkaline HER.** LSV curves of PBA‑350 before and after extended stability testing, confirming minimal performance loss and preserved kinetics.

**Figure S38.** **Elemental stability during alkaline HER.** Atomic fractions of Fe, Mn, Co and Ni in PBA‑350 before and after HER stability testing in 1.0 M KOH, indicating limited leaching and preserved composition.

**Figure S39. Post-HER microscopy of PBA‑350.** HAADF‑STEM images and STEM‑EDS elemental maps of PBA‑350 after HER stability testing in 1.0 M KOH, showing retention of the nanocage morphology and the Ni/PBA interfacial architecture.

**Figure S40.** **Post-HER surface chemistry from XPS.** High-resolution XPS spectra of (A) Fe 2p, (B) Co 2p, (C) Ni 2p and (D) Mn 2p for PBA‑350 after HER in 1.0 M KOH, revealing partial surface reoxidation/rehybridization while preserving the defect–metal motif.

**Figure S41.** **Operando Raman under HER potentials.** Operando Raman spectra of PBA‑350 recorded at different potentials (vs RHE), probing potential-dependent changes in interfacial water and surface species linked to Volmer water dissociation.

**Figure S42.** **Operando Bode response of the pristine PBA.** Bode phase plots of PBA‑RT measured at different potentials, serving as an interfacial-kinetics baseline for the vacancy–exsolution-engineered PBA‑350.

**Figure S43. Potential-dependent phase maxima from operando EIS.** Phase-peak angles for PBA‑RT and PBA‑350 as a function of applied potential, extracted from operando Bode phase plots and reflecting accelerated interfacial dynamics for PBA‑350.

**Figure S44. Atomistic models used for mechanistic calculations.** Theoretical structures of PBA, vacancy-containing PBA (PBA‑V_CN_) and Ni-decorated vacancy-containing PBA (Ni/PBA‑V_CN_) used in DFT and MD simulations to isolate the roles of vacancies and Ni exsolution.

**Figure S45.** **Vacancy-induced charge redistribution.** Charge-density difference maps for the PBA and PBA‑V_CN_ models (isosurface 0.05 e Å⁻³), highlighting electron accumulation/depletion introduced by CN vacancies.

**Figure S46.** **Interfacial polarisation and electrostatic potential.** (A) Plane-averaged charge-density difference along the surface normal for PBA, PBA‑V_CN_ and Ni/PBA‑V_CN_, showing enhanced interfacial polarisation upon vacancy formation and Ni loading. (B) Corresponding electrostatic potential profiles evidencing an internal electric field that promotes charge transfer to adsorbates.

**Figure S47**. **Electronic structure and p–d coupling in PBA-derived models.** Projected density of states (PDOS) for (A) PBA, (B) PBA‑V_CN_ and (C) Ni/PBA‑V_CN_, illustrating vacancy- and Ni-induced enhancement of states near E_F_ and strengthened d–d and p–d coupling, consistent with improved interfacial charge transport and optimised intermediate binding.

**Figure S48**. **CN-2p states and p-band centre descriptor.** PDOS of CN-2p orbitals and extracted p-band centres for PBA, PBA‑V_CN_ and Ni/PBA‑V_CN_, quantifying vacancy- and Ni-driven tuning of metal–ligand covalency.

**Figure S49**. **H_2_O adsorption energetics on candidate active sites.** Calculated H₂O adsorption energies on representative Ni and Co sites in PBA, PBA‑V_CN_ and Ni/PBA‑V_CN_, showing strengthened water binding at vacancy-stabilised Ni sites.

**Figure S50**. **Electronic signatures of adsorbed water on Ni sites.** p‑PDOS of the O atom in adsorbed H₂O on various Ni sites (left) and corresponding 2D charge-density difference maps after adsorption (right; ±0.5 e Å⁻³), revealing enhanced Ni–H₂O interaction in Ni/PBA‑V_CN_.

**Figure S51**. **OH^−^ desorption energetics on Co sites.** Calculated OH⁻ desorption free energies on representative Co sites, indicating facilitated OH* removal in the vacancy–exsolution-engineered interface.

**Figure S52. Free-energy landscape for hydrogen evolution and the proposed mechanism diagram.** (A) Free-energy diagram for H* formation/evolution on different sites, highlighting near-thermoneutral ΔG_H*_ at the Ni/PBA‑V_CN_ interface. (B) ΔG_H*_ plotted against ICOHP for Ni–H and Co–H configurations, establishing a bonding–activity relationship consistent with a dual-site mechanism. (c) Schematic illustration of the proposed alkaline HER pathway on the Ni/PBA-VCN interface. The five panels show the sequential reaction states: (I) initial active site, (II) H_2_O adsorption, (III) H_2_O dissociation with H* formation, (IV) *H_2_ formation through the Heyrovský step, and (V) desorption of gaseous H_2_ from the catalytic surface. The red and white balls refer to the oxygen and hydrogen atom.

**Figure S53. Post-AEM electrolyser surface chemistry.** High-resolution XPS spectra of (A) Fe 2p, (B) Co 2p, (C) Ni 2p and (D) Mn 2p for PBA‑350 after AEM electrolyser operation, assessing chemical stability under device-relevant conditions.

**Figure S54. Elemental stability during AEM operation.** Atomic fractions of Fe, Mn, Co and Ni in PBA‑350 before and after AEM electrolyser stability testing in 1.0 M KOH.

**Figure S55.** **Seawater HER kinetics and charge-transfer diagnostics.** (a) Tafel plots and (b) Nyquist plots in 1.0 M KOH + 0.5 M NaCl, showing that PBA‑350 retains accelerated kinetics and low charge-transfer resistance in simulated seawater.

**Figure S56.** **Operando Bode response in simulated seawater.** Bode plots of PBA‑RT under various applied potentials in 1.0 M KOH + 0.5 M NaCl, providing a seawater baseline for interfacial kinetics.

**Figure S57. Overall water-splitting performance in alkaline and simulated seawater electrolytes.** Polarization curves of PBA‑350‖PBA‑350 and Pt/C‖IrO₂ electrolysers in 1.0 M KOH and 1.0 M KOH + 0.5 M NaCl, benchmarking device performance under saline conditions.

**Figure S58. Post-seawater surface chemistry from XPS.** High-resolution XPS spectra of (A) Fe 2p, (B) Co 2p, (C) Ni 2p and (D) Mn 2p for PBA‑350 after stability testing in 1.0 M KOH + 0.5 M NaCl.

**Figure S59.** **Elemental stability during simulated seawater operation.** Atomic fractions of Fe, Mn, Co and Ni in PBA‑350 before and after stability testing in 1.0 M KOH + 0.5 M NaCl.

**Figure S60. Interfacial ion and water distributions from MD simulations.** Spatial distributions of Na⁺, K⁺, Cl⁻, OH⁻ and H₂O near PBA, PBA‑V_CN_ and Ni/PBA‑V_CN_ surfaces, showing salt-ion exclusion and enhanced interfacial hydration at Ni/PBA‑V_CN_.

**Figure S61. Interfacial water enrichment at Ni/PBA‑VCN**. Number of H₂O molecules within 5 Å of the electrode surface for PBA, PBA‑VCN and Ni/PBA‑VCN, quantifying the denser protective water layer that correlates with seawater tolerance.

**Supplementary Tables**

**Table S1.** **Benchmarking of alkaline HER activity.** Comparison of overpotentials at 10 mA cm⁻² and Tafel slopes for representative HER catalysts in 1.0 M KOH, positioning PBA‑350 among state-of-the-art systems.

| **Catalyst** | **Overpotential, mV**  (at 10 mA cm^-2^) | **Tafel slope**  (mV dec^-1^) | **Ref.** |
| --- | --- | --- | --- |
| **Transition metal-based** |  |  |  |
| Co/MoN | 52 | 77.5 | 1 |
| Ni-Ni(OH)_2_ | 72 | 43 | 2 |
| sc-Ni_2_P/NiHO/NF | 85 | 75 | 3 |
| FeP nanosheet | 95 | 64 | 4 |
| CoMoC | 46 | 46 | 5 |
| MnSe_2_/CP | 118 | 132.8 | 6 |
| WN-NiN/CFP | 36.8 | 61.3 | 7 |
| CoMoC | 55 | 87 | 8 |
| Co/CoMoN/NF | 61 | 68.9 | 9 |
| Ni/CeO_2_@N-CNFs | 100 | 85.7 | 10 |
| **Nobel-metal based** |  |  |  |
| d-PtSe_2_ | 59 | 88 | 11 |
| Ru/RuO_2_ | 17 | 35 | 12 |
| Pt−Co(OH)_2_/CC | 32 | 70 | 13 |
| Rh_2_Sb NBs/C | 28.3 | 24.6 | 14 |
| Ru/NC-10 | 15.8 | 34.6 | 15 |
| Ru_NPs_-RuCr_APs_ | 31 | 53 | 16 |
| c-RP/IP HNTs | 23.2 | 30.7 | 17 |
| Rh NSs | 37.8 | 98.3 | 18 |
| Rh_2_P | 30 | 50 | 19 |
| Mo_2_C@NC@Pt | 47 | 57 | 20 |
| PBA-350 | 28.4 | 56.4 | **This work** |

**Table S2**. **Turnover frequency analysis in simulated seawater**. TOF values of Pt/C, PBA-RT and PBA-350 in 1.0 M KOH + 0.5 M NaCl. The comparison shows that PBA-350 exhibits intrinsic HER kinetics close to Pt/C and superior to PBA-RT under simulated seawater conditions.

| **Catalysts** | **TOF (s^-1^)** |
| --- | --- |
| Pt/C | 1.53 |
| PBA-RT | 0.58 |
| PBA-350 | 1.36 |

**References**

[1] Sun, J. et al. Co/MoN hetero-interface nanoflake array with enhanced water dissociation capability achieves the Pt-like hydrogen evolution catalytic performance. *Appl. Catal. B* **286**, 119882 (2021).
[2] Zhong, W. et al. Interfacial electron rearrangement: Ni activated Ni(OH)_2_ for efficient hydrogen evolution. *Journal of Energy Chemistry* **61**, 236–242 (2021).
[3] You, B. et al. Negative Charging of Transition-Metal Phosphides via Strong Electronic Coupling for Destabilization of Alkaline Water. *Angew. Chem. Int. Ed.* **58**, 11796–11800 (2019).
[4] Zhao, X. et al. Elucidating the sources of activity and stability of FeP electrocatalyst for hydrogen evolution reactions in acidic and alkaline media. Appl. Catal. B 260, 118156 (2020).
[5] Liu, G. et al. A highly efficient alkaline HER Co–Mo bimetallic carbide catalyst with an optimized Mo d-orbital electronic state. *J. Mater. Chem. A* **7**, 12434-12439 (2019).
[6] Roy, K. et al. Dimensionality-Tailored Ferromagnetism in Quasi-Two-Dimensional MnSe2 for the Magnetoelectrochemical Hydrogen Evolution Reaction in Alkaline Media. *ACS Nano* **18**, 35, 24569–24580 (2024).
[7] Yang, L. et al. Customizing Bonding Affinity with Multi-Intermediates via Interfacial Electron Capture to Boost Hydrogen Evolution in Alkaline Water Electrolysis. *Angew. Chem. Int. Ed.* **64**, e202414518 (2025).
[8] Fu, H. et al. Hydrogen Spillover-Bridged Volmer/Tafel Processes Enabling Ampere-Level Current Density Alkaline Hydrogen Evolution Reaction under Low Overpotential. *J. Am. Chem. Soc.* **144**, 13, 6028–6039 (2022).
[9] Ma, H. et al. Interface Engineering of Co/CoMoN/NF Heterostructures for High-Performance Electrochemical Overall Water Splitting. *Adv. Sci.* **9**, 2105313 (2022).
[10] Li, T. et al. Manipulation of Mott−Schottky Ni/CeO_2_ Heterojunctions into N-Doped Carbon Nanofibers for High-Efficiency Electrochemical Water Splitting. *Small* **18**, 2106592 (2022).
[11] Chang, Y. et al. Excellent HER and OER Catalyzing Performance of Se-Vacancies in Defects-Engineered PtSe_2_: From Simulation to Experiment. *Adv. Energy Mater* **12**, 2102359 (2022).
[12] Dang, Y. et al. Partially reduced Ru/RuO_2_ composites as efficient and Ph-universal electrocatalysts for hydrogen evolution. *Energy Environ. Sci.* **14**, 5433-5443 (2021).
[13] Xing, Z. et al. Ultrafine Pt Nanoparticle-Decorated Co(OH)_2_ Nanosheet Arrays with Enhanced Catalytic Activity toward Hydrogen Evolution. *ACS Catal.* **7**, 7131–7135 (2017).
[14] Zhang, Y. et al. Atomically isolated Rh sites within highly branched Rh_2_Sb nanostructures enhance bifunctional hydrogen electrocatalysis. *Adv. Mater.* **33**, 2105049 (2021).
[15] Baek, J. et al. Dominant Role of Coexisting Ruthenium Nanoclusters Over Single Atoms to Enhance Alkaline Hydrogen Evolution Reaction. *Adv. Sci.* **12**, 2414012 (2025).
[16] Eskandari, P. et al. Enhanced Hydrogen Evolution Reaction in Alkaline Media via Ruthenium–Chromium Atomic Pairs Modified Ruthenium Nanoparticles. *Adv. Mater.* 2419360 (2025).
[17] Hong, Y. et al. Ru_2_P/Ir_2_P Heterostructure Promotes Hydrogen Spillover for Efficient Alkaline Hydrogen Evolution Reaction. *Adv. Energy Mater.* **14**, 2401426 (2024).
[18] Zhang, Z. et al. Evoking ordered vacancies in metallic nanostructures toward a vacated Barlow packing for high-performance hydrogen evolution. *Sci. Adv.* **7**, eabd6647 (2021).
[19] Yang, F. et al. A Monodisperse Rh_2_P-Based Electrocatalyst for Highly Efficient and pH-Universal Hydrogen Evolution Reaction. *Adv. Energy Mater.* **8**, 1703489 (2018).
[20] Chi, J. et al. N‑Doped Sandwich-Structured Mo_2_C@C@Pt Interface with Ultralow Pt Loading for pH-Universal Hydrogen Evolution Reaction. *ACS Appl. Mater. Interfaces* **11**, 4047−4056 (2019).
